# Supplementary material for: Nanoscale cooperative adsorption for materials control
Source: Nat Commun. 2021 Jul 13;12:4287. doi: 10.1038/s41467-021-24590-y (PMC8277846; doi:10.1038/s41467-021-24590-y)
Supplement: Supplementary file 1 — Supplementary Information [file 41467_2021_24590_MOESM1_ESM.pdf]

Supplementary Information

Nanoscale cooperative adsorption for materials control

Rong Ye[†], Ming Zhao[†], Xianwen Mao, Zhaohong Wang, Diego A. Garzón, Heting Pu, Zhiheng Zhao, Peng Chen*

[†]These authors contributed equally to this work.

*Correspondence to: pc252@cornell.edu

Table of Contents

1 Supplementary Materials and methods 3
1.1 General chemicals and instruments 3
1.2 Synthesis and characterization of mesoporous-silica-coated Au nanoplates 3
1.2.1 Synthesis and morphology characterization of Au nanoplates via electron microscopy 3
1.2.2 Mesoporous silica shell coating, thickness characterization, and subsequent ligand removal........ 3
1.2.3 The necessities and advantages of the mesoporous silica shell 5
1.3 Synthesis and characterization of mesoporous-silica-coated Au nanorods........ 6
1.3.1 Synthesis and morphology characterization of Au nanorods via electron microscopy........ 6
1.3.2 Preparation and characterization of mesoporous-silica-coated Au nanorods and ligand removal........ 7
1.4 Electrochemical UPD of Pb on Au nanoparticles and confirmation of facet assignments of Au nanoplates and nanorods........ 7
1.5 Hill model of cooperative adsorption and the equation derivation for analyzing COMPEITS titration comprising cooperative adsorption of the competitor 8
1.6 Bulk measurements confirm the Langmuir-Hinshelwood kinetics of the fluorogenic auxiliary reaction, in which the reactant resazurin adsorbs noncooperatively 10
1.7 Single-molecule fluorescence imaging experiments........ 11
1.8 Single-molecule fluorescence image analysis 12
1.8.1 Single-molecule fluorescence image analysis for super-resolution localization........ 12
1.8.2 Quantitative single-molecule counting algorithm to correct for over-counting and underestimation of product molecules 14
1.8.3 Overlay of SEM and optical microscopy (OM) images 15
1.8.4 Obtaining v_R for a whole particle or different sub-particle sections 16
1.9 Facet-controlled synthesis of colloidal Au nanoparticles in the presence of increasing [CTAB] and their SEM characterization: transition from irregular-shaped nanoparticles to high-quality nanoplates 17
2 Supplementary bulk reaction titration confirms: (1) CTAB/CTAOH/CTAC adsorb with positive cooperativity; (2) PVPs adsorb with negative cooperativity; (3) I^-/Br^-/BME adsorb non-cooperatively; (4) monomeric VP, EtOH, and K^+ have negligible adsorption; (5) [NH_2OH] is saturated for the fluorogenic reaction kinetics 18
3 Supplementary results of single-molecule reaction imaging and COMPEITS imaging of single 5-nm Au nanoparticles 20
3.1 Super-resolution images of fluorogenic auxiliary reaction and COMPEITS images of ligand adsorption........ 20
3.2 Possible residual citrate in solution does not affect the results from 5-nm Au nanoparticles 22
3.3 Decrease of reaction rates during COMPEITS titration is not due to catalyst deactivation........ 23
3.4 Ligand adsorption titration curves: adsorption affinity and (non)cooperativity of CTAB/CTAOH/CTAC, PVPs, halides, and thiol 23
4 Supplementary results of COMPEITS imaging of ligand adsorption on single Au nanoplates........ 25
4.1 COMPEITS images indicate spatially (in)homogeneous adsorption on single nanoplates of different ligands 25
4.2 The decrease in reaction rates during COMPEITS titration is not due to catalyst deactivation........ 26
4.3 Additional statistical plots of K and h of ligand adsorption on single nanoplates reveal sub-particle and sub-facet differences 27
4.4 Particle-averaged titration analyses also identify sub-particle differences in ligand adsorption........ 29
4.5 Adsorption strength and cooperativity vs. nanoplate size and shape 31
5 Supplementary results of COMPEITS imaging of ligand adsorption on single Au nanorods 33
5.1 Additional statistical plots of K and h of ligand adsorption on single nanorods reveal sub-particle and sub-facet differences 33
5.2 Particle-averaged titration analyses also identify sub-particle differences in ligand adsorption........ 34
5.3 Adsorption strength and cooperativity vs. nanorod size 36
5.4 Contributions of under-coordinated atoms are insignificant compared with facet orientations 38
6 Supplementary control experiments and discussions on facet-controlled synthesis of Au nanoparticles demonstrating the crossover behavior of ligand adsorption 38
6.1 Ascorbic acid/ascorbate adsorption onto Au particles are likely insignificant in the presence of CTAB 38
6.2 Potential contribution of Au species adsorption in the facet-controlled Au nanoparticle synthesis........ 39
6.3 The CTAB concentrations in Au nanoparticle syntheses are all below the critical micelle concentration at the reaction temperature 40
6.4 Possible reasons for the existence of crossover behavior of CTAB adsorption on Au{110} vs. Au{111} 40
6.5 Predicting the crossover concentration c_x........ 41
6.6 Potential broader applications of the crossover behavior of ligand adsorption........ 41
6.7 Predicting relative multi-layer adsorption trends........ 42
6.8 The crossover behavior in our shape-controlled synthesis of Au nanoparticles should not be caused by the seeding effect.... 42
7 Supplementary references........ 42

1 Supplementary Materials and methods

1.1 General chemicals and instruments

3-mercaptopropyltrimethoxysilane (MPTMS, 95%, 175617), tetraethylorthosilicate (TEOS, 98%, 131903), sodium borohydride ($NaBH_4$, 99%, 213462), L-ascorbic acid (≥99.0%, A1417), polyvinylpyrrolidone (average M_w ~55,000, 856568), polyvinylpyrrolidone (average M_w 40,000, PVP40), polyvinylpyrrolidone (average M_w 10,000, PVP10), 2-mercaptoethanol (BME, ≥99.0%, M6250), cetyltrimethylammonium chloride solution (CTAC, 25 wt. % in H_2O, 292737), hexadecyltrimethylammonium hydroxide solution (CTAOH, 10 wt. % in H_2O, 439231), potassium bromide (KBr, anhydrous, ≥99.9% trace metals basis, 449970), lead nitrate ($Pb(NO_3)_2$, ≥99.0%, 228621), and hydroxylamine hydrochloride ($NH_2OH{\cdot}HCl$, 99%, 159417) were purchased from Sigma-Aldrich. Sodium silicate nonahydrate (S25567), sodium hydroxide (Pellets, S318), sodium citrate (Na_3Cit, S279-500), acetone (A19-1), and absolute ethanol (200 proof, BP2818-4) were purchased from Fisher Scientific. Other chemicals included hydrogen tetrachloroaurate ($HAuCl_4$, 99.999%, Beantown Chemical, 131445), cetyltrimethylammonium bromide (CTAB, 99+%, Acros Organics, 22716), potassium iodide (KI, 99.5%, Fluka, 60399), Gold colloid (5 nm, Ted Pella, 15702). All chemicals above were used without further treatment unless otherwise noted. Lemon grass was purchased from Wegmans store in Ithaca, NY, and thoroughly cleaned by water. Resazurin sodium salt (Molecular Probes, Thermo Fisher Scientific, R12204) was purified via thin layer chromatography before use. All H_2O used was purified via an Elga water purification system to reach the resistivity of 18.2 MΩ/cm.

The UV-Vis absorption spectra were obtained with a Beckman Coulter DU 800 spectrometer and the fluorescence emission spectra were recorded on a Varian Cary Eclipse fluorescence spectrometer. Transmission electron microscopy (TEM) was performed either on a FEI F20 TEM STEM operated at 200 kV or FEI Tecnai Spirit Twin operated at 120 kV at the Cornell Center for Materials Research (CCMR). Zeiss Gemini 500 Scanning Electron Microscope (SEM) operated at 10~15 keV at CCMR was used to capture SEM images. COMPEITS imaging experiments were carried out on a home-built microscope (see 1.6).

1.2 Synthesis and characterization of mesoporous-silica-coated Au nanoplates

1.2.1 Synthesis and morphology characterization of Au nanoplates via electron microscopy

Au nanoplates were synthesized following a procedure modified from a previous report[1,2]. Typically, 10 g of cleaned and finely cut lemon grass was boiled with 50 mL water for 6 min. After removing the solids via centrifugation at 3000 g for 6 min, the supernatant was mixed with 200 mL of 1 mM $HAuCl_4$ and then allowed to be shaken at 0.5 Hz at room temperature overnight. The products were collected via centrifugation at 3000 g, followed by washing with water for three cycles. The morphology and shape yield of the sample were examined by TEM. Supplementary Fig. 1a shows TEM images of as-synthesized Au nanoplates, which exhibit triangular and hexagonal shapes. On average, the radius (the mean distance from the center to the vertex) of the nanoplates is 0.93 ± 0.34 μm (Supplementary Fig. 1d) from the 236 nanoplates imaged in this work, and Supplementary Fig. 1e shows the shape distribution of these nanoplates. The thickness of such Au nanoplates is 14 ± 1 nm from atomic force microscopy in our previous work[2].

Earlier structural characterizations by multiple groups showed that the nanoplates were oriented with {111} planes as their basal planes and bounded by {110} planes at the edges[3-6]. Such facet assignment is also confirmed by electrochemical underpotential Pb deposition that resolved the deposition potentials on the two respective facets (see Section 1.4 later and Supplementary Fig. 2a).

1.2.2 Mesoporous silica shell coating, thickness characterization, and subsequent ligand removal

The as-synthesized Au nanoplates were then coated with mesoporous silica in three major steps as previously reported[2,7-10]: (I) coating the particles with a thin silica layer, following the Ströber method[9]; (II)

further growth of the silica layer to a shell of a desired thickness; (III) etching the silica shell to make it mesoporous. Briefly, for Step I, Au nanoparticles dispersed in water were diluted to 30 mL with water and was mixed with 7.5 μL of freshly prepared 20 mM 3-mercaptopropyltrimethoxysilane (MPTMS) in acetone while stirring vigorously. After 30 min, 1 mL of freshly prepared aqueous solution of 0.54% w/v Na_2SiO_3 (pH 10-11) was added dropwise and kept stirring for 48 h at room temperature. Afterwards, the reaction mixture was centrifuged at 1000 g for 20 min to precipitate the nanoparticles. In Step II, the Au nanoparticles were re-suspended in 30 mL EtOH/H_2O mixture (2.5:1 v/v), to which 350 μL of 0.1 M NaOH was added followed by 30 μL of tetraethylorthosilicate (TEOS). The mixture was stirred for at least 1 d at room temperature. The resulting Au nanoparticles were collected via centrifugation at 1000 g for 10 min. In Step III, the silica-coated nanoparticles were re-suspended in 20 mL H_2O/EtOH mixture (4:1 v/v) saturated with CTAB. 150 μL of 0.1 M NaOH was added and stirred at room temperature for 15 min. The solution was heated in a 70 °C water bath for ~2 h. The mesoporous-silica-coated Au (Au@mSiO_2) nanoparticles were collected after centrifugation at 1000 g for 10 min, followed by washing with water for at least three times. Supplementary Fig. 1b-c are representative TEM and SEM image of Au@mSiO_2 nanoplates after washing, respectively. The average thickness of the mesoporous silica shell is 39 ± 6 nm (Supplementary Fig. 1f). Based on this method, the mesoporous silica shells have NaOH-etched pores with an average pore size of ~35 Å[8], which enables reactants and products to freely diffuse in and out of these pores.

The organic ligands bound to the Au surface, including CTAB, were removed by UV-ozone treatment before imaging studies, following literature procedures[2,11]. Briefly, the washed Au@mSiO_2 nanoplates were dispersed on a quartz slide, dried, and placed ~2 cm below a UV lamp (UVP Pen-Ray 90-0012-01 Model 11SC-1 Mercury UV Lamp, 254 nm Longwave) in air for about 12 hours.

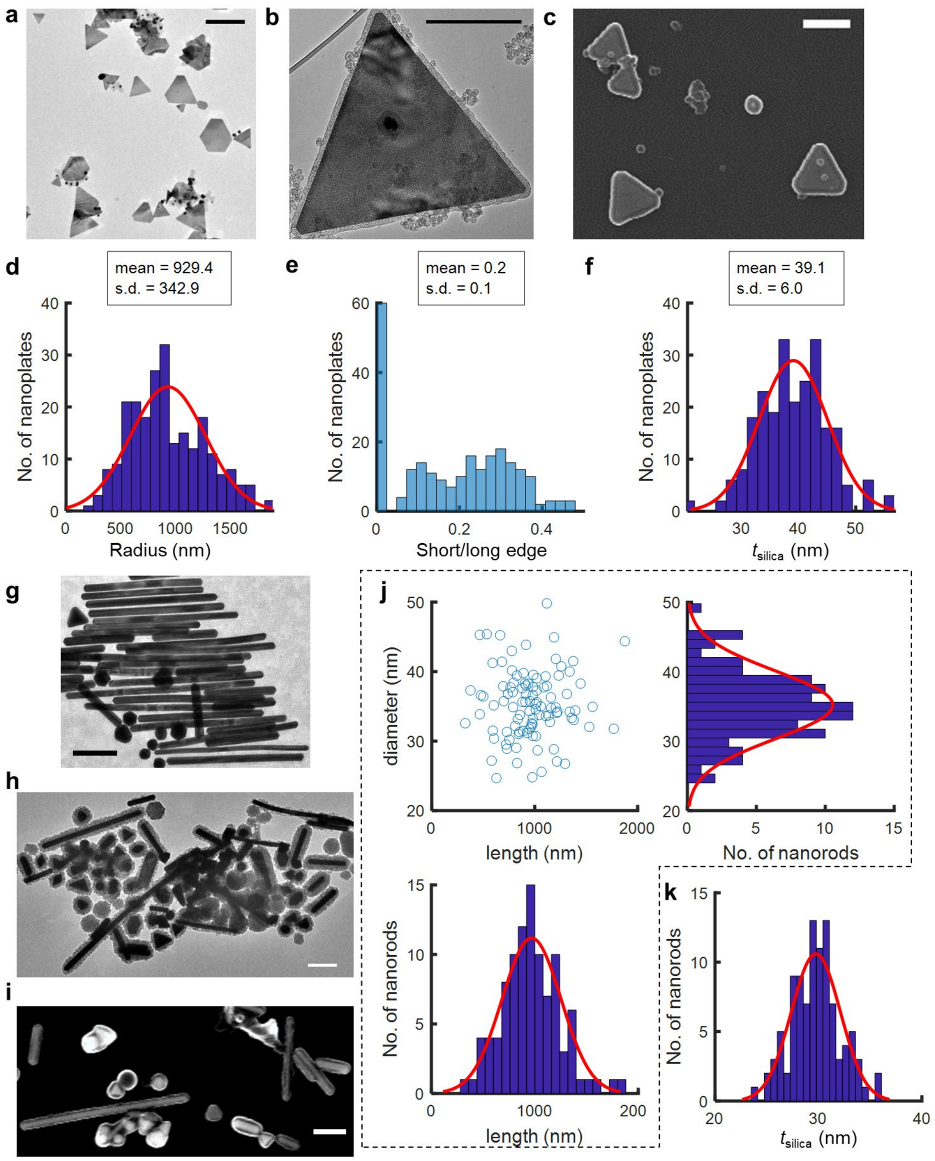

Supplementary Fig. 1 | Electron microscopy characterizations of Au nanoplates and nanorods. a-b, Representative TEM images of Au nanoplates, as-synthesized (**a**) and after coating with mesoporous silica (**b**). **c**, Representative SEM image of mesoporous-silica-coated Au nanoplates. Samples in (**b-c**) were before UV-ozone treatment. Scale bars are 500 nm in (**a-c**). **d-f**, Distribution of the radius, i.e., the averaged distance from the center to the vertex (**d**), short/long edge length ratio, which is 0 for a triangle and 1 for an equilateral hexagon (**e**), and the thickness of the mesoporous silica shell t_{silica} (**f**) of the 236 nanoplates imaged in this work. Red lines in **d** and **f** are Gaussian fits. **g-h**, Representative TEM images of Au nanorods, as-synthesized (**g**) and after coating with mesoporous silica (**h**). **i**, Representative SEM image of mesoporous-silica-coated Au nanorods. Samples in **h-i** were before UV-ozone treatment. Scale bars are 200 nm in **g-i**. **j**, Correlation of the length and the diameter (the width) of the 100 nanorods chosen for COMPEITS analysis, along with the histograms. Red lines are Gaussian fits. The average length is 0.97 ± 0.29 μm, and the average diameter is 35 ± 5 nm. **k**, The thickness of the mesoporous silica shell t_{silica} of the corresponding nanorods. The average thickness is 30 ± 2 nm.

1.2.3 The necessities and advantages of the mesoporous silica shell

The mesoporous silica shell on the Au nanoplates or nanorods (see later) offers a number of benefits for both the single-molecule catalysis imaging experiments and the catalytic activity study:

1) The mesoporous silica shell enables the stabilization and dispersion of Au nanoparticles in solution upon the removal of their surface organic capping ligands. Organic ligands such as CTAB are

involved in the preparation of these Au nanoparticles for stability, dispersion, and shape control in the solution. It is essential to remove these ligands for the clean measurement of the adsorption interaction between the Au nanoparticles and the capping ligands including CTAB.

2) These organic ligands passivate the surface of the nanoparticles and lower their catalytic activity. Without the mesoporous silica shell, the nanoparticles would aggregate after the ligand removal (e.g., via UV-Ozone treatment; see section 1.2.2), and also be difficult to be re-dispersed. Thus, the silica shell facilitates the removal of organic ligands for the high catalytic activity necessary for the COMPEITS experiments.
3) The mesoporous silica shell can also *temporarily* trap the catalytically produced fluorescent probe molecules (i.e., resorufin) near the surface of the Au nanoparticles. These probe molecules are trapped inside the shell nearby the locations where they were catalytically generated, enabling the detection of their production locations (active sites) at the single-molecule level before they diffuse away into the surrounding solution.
4) The mesoporous silica shell allows for the detection of fluorescent probe molecules away from the metallic surface of the nanoparticles, where the fluorescence quenching by the Au surface might impede the imaging. In our experience, detecting the fluorescence of resorufin directly on the surface of these nanoplates and nanorods of such sizes is problematic.
5) The mesoporous silica shell stabilizes the nanoparticles morphology during the catalysis imaging. Nanoparticles, especially those with well-defined facets, are known to be susceptible to surface restructuring in the catalytic process. The shell covers the surfaces of Au nanoparticles and increases the stability of their morphology and crystallographic orientation during catalytic reactions. Consistently, we did not observe discernible morphology changes in these Au@mSiO_2 nanoparticles after the catalysis imaging[2,7].

Many evidences support that the mesoporous silica shell has insignificant effect on the adsorption cooperativity trends observed in our measurements:

1) The cooperative adsorption for CTAB (and PVP) occurs both in the absence (5-nm Au nanoparticles) and in the presence (nanoplates and nanorods) of the shell. Therefore, this shell does not render cooperativity.
2) The magnitude of h for CTAB adsorption on 5-nm Au nanoparticles, Au nanoplates, and Au nanorods are all roughly 2 (or all roughly 0.7 for PVP). This similarity between the naked 5-nm Au nanoparticles and mesoporous-silica-coated nanoplate and nanorods further confirm that the silica shell does not render the measured cooperativity.
3) We persistently observed the anti-correlation between affinity and cooperativity for CTAB/PVP adsorption on 5-nm Au nanoparticles (without a silica shell) and nanoplates/nanorods (coated with mesoporous silica).
4) The {111} facet shows stronger cooperativity than the {110} facet, regardless of whether the {111} facet is located dominantly at low curvature regions (i.e., the top flat facet on nanoplates) or at high curvature regions (i.e., at the tips of nanorods) of the particle. Therefore, the presence of the mesoporous silica shell should not alter h biasedly or change the trends across regions.

1.3 Synthesis and characterization of mesoporous-silica-coated Au nanorods

1.3.1 Synthesis and morphology characterization of Au nanorods via electron microscopy

Penta-twinned Au nanorods were synthesized in a three-step seed-mediated growth method following the literature[12]. Briefly, (A) Seeds@Citrate solution: At room temperature, 100 μL of 50 mM $HAuCl_4$ was added to 20.0 mL of 0.25 mM Na_3Cit. Next, 600 μL of a freshly prepared 100 mM $NaBH_4$ solution was rapidly injected under vigorous stirring (>1400 rpm). After 2 min the solution was kept under mild stirring (400 rpm) for 40 min at room temperature and for 15 min at 40-45 °C before use. (B) Seeds@CTAB solution: 12.5 μL of 50 mM $HAuCl_4$ was added to a mixture of 3 mL of water and 2 mL of

0.1 M CTAB. The solution was heated to over 30 °C to facilitate the dissolution of CTAB and then cooled down to 22 °C before use. 12.5 μL of 0.1 M AA was then added to the solution and shaken by hand; the mixture turned colorless in a few seconds. Finally, 835 μL of the Seeds@Citrate solution was added, shaken by hand and left undisturbed for 3 hours at 22 °C. (C) Growth solution: CTAB (4 mL 0.1 M) was added to 46 mL of water. 0.125 mL of 0.05 M $HAuCl_4$ solution was then added; the solution was gently shaken and cooled down to 22 °C. Subsequently, 0.156 mL of 0.1 M AA solution was added to the mixture, and the solution was gently shaken until it turned completely colorless. Finally, 65 μL Seeds@CTAB was added to the growing mixture; the solution was vigorously shaken by hand and then left undisturbed overnight at 20 °C. The resultant nanorods in solution, purple in color, were centrifuged at 300 g for 20 min and washed in ethanol, then water for three times. The morphology and shape yield of the nanoplates and nanorods were examined by TEM.

Supplementary Fig. 1g is a representative TEM image of the as-synthesized Au nanorods. Although the sample inevitably contained particles in other shapes, only nanorods longer than 100 nm visualized in SEM were chosen for further data analysis due to the spatial resolution of single-molecule super-resolution imaging (see later). Supplementary Fig. 1j shows the correlations of the length and the diameter (the width) of 100 nanorods analyzed for COMPEITS measurements, averaging at 0.97 ± 0.29 μm in length and 35 ± 5 nm in diameter.

For penta-twinned nanorods, the tips were consistently assigned as having {111} facets, but the sides were assigned as {110} facets by El-Sayed et al. and Harmer et al.[13,14], or {100} facets by Mann et al.[15], even though Harmer et al.[14] and Mann et al.[15] followed the same synthesis procedure by Murphy et al.[16]. El-Sayed et al.[13] suggested that the higher energy {110} facets showed reconstruction into more stable {100} facets. Our cyclic voltammetry (CV) measurements of electrochemical underpotential deposition (UPD) of Pb on these Au nanorods (Section 1.4; Supplementary Fig. 2b) confirm that the sides are enclosed by {110} facets, which are higher in energy and have lower surface atom packing density than the tips' {111} facets.

1.3.2 Preparation and characterization of mesoporous-silica-coated Au nanorods and ligand removal

Our preparation of mesoporous-silica-coated Au nanorods follows the same protocol as the one for Au nanoplates. Supplementary Fig. 1h-i are representative TEM and SEM image of mesoporous-silica-coated Au nanorods, respectively. The average thickness of the mesoporous silica shell is 30 ± 2 nm (Supplementary Fig. 1k). These nanorods also went through the UV-Ozone treatment for the removal of organic ligands before use, in the same way as the nanoplates.

1.4 Electrochemical UPD of Pb on Au nanoparticles and confirmation of facet assignments of Au nanoplates and nanorods

The UPD of Pb on Au nanoparticles were carried out in a three-electrode cell using an electrochemical workstation (CHI 1200a potentiostat) following literature[17,18] (see Methods). Supplementary Fig. 2a shows the CV curve of the as-synthesized Au nanoplates (Supplementary Fig. 1a). Compared with the two small peaks at 0.53 & 0.67 V, the dominant peaks at 0.38 & 0.45 V suggest the prevalence of Au{111}, consistent with the feature of Au nanoplates whose large flat surfaces are {111} facets while the small side edges are {110} facets. Supplementary Fig. 2b shows the CV curve of Au nanorods (Supplementary Fig. 1g). The pair of peaks representing Au{110} located at 0.53 & 0.67 V are dominant in the curve, in agreement with and confirming that the side facets along the length of Au nanorods are {110} facets. The presence of Au{111} feature in the CV of Au nanorods can be attributed to: *i*) the {111}-enclosed penta-twinned structure at the two tips of Au nanorods; and *ii*) the presence of a small portion of {111}-dominant Au nanoplates and spheres in the sample (see Supplementary Fig. 1g).

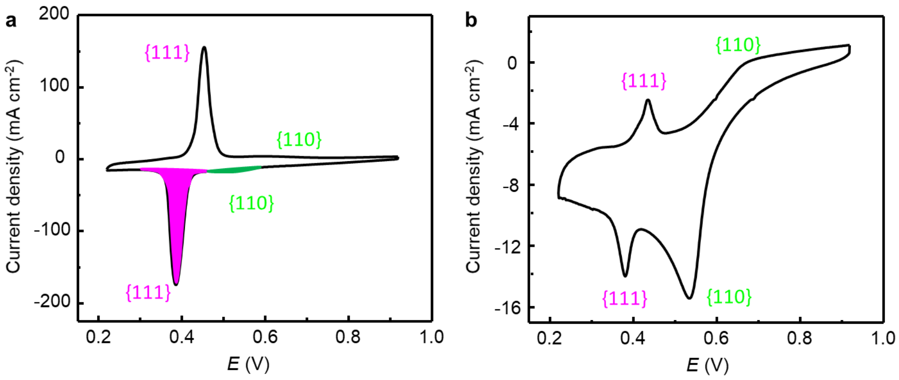

Supplementary Fig. 2 | CV curves of Pb UPD on Au nanoplates (a) and nanorods (b), respectively. The pronounced peaks at 0.38 & 0.45 V and 0.53 & 0.67 V in **a** and **b** suggest the dominance of Au{111} and Au{110} facets in nanoplates and nanorods, respectively. The integrated deposition peak areas for Au{111} and Au{110} are represented by magenta and green colors, respectively, in **a**.

1.5 Hill model of cooperative adsorption and the equation derivation for analyzing COMPEITS titration comprising cooperative adsorption of the competitor

We consider resazurin (R) and a ligand L competitively adsorb on the same type of surface sites on the catalyst surface, in which L can adsorb cooperatively and R follows the noncooperative Langmuir adsorption (Section 1.6 later).

First, we consider the Hill cooperative adsorption of the ligand L on a cluster of h adsorption sites, e.g., a patch of surface having h sites, in the absence of R. Note each adsorption site could comprise one or more surface metal atoms, depending on the adsorption geometry of a particular ligand and on the particular metal surface structure (e.g., different facets). We follow the all-or-none approximation used in the classic Hill model[19,20], i.e., the cluster is either bound by h ligands as M_h-L_h, or completely free of L, as shown in the chemical equation:

$$\mathrm{M}_h\text{-}\mathrm{L}_h \leftrightarrow \mathrm{M}_h + h\mathrm{L}$$

By definition, the dissociation equilibrium constant K_d follows

$$K_\mathrm{d} = \frac{[\mathrm{M}_h][\mathrm{L}]^h}{[\mathrm{M}_h\text{-}\mathrm{L}_h]} = (1/K_\mathrm{L})^h \qquad \text{Eq. S1}$$

where $[M_h]$ and $[M_h\text{-}L_h]$ are the concentrations of free and occupied adsorption clusters, respectively; K_L is the inverse $K_{0.5}$ seen in some textbooks[21] and has the inverse concentration unit, and can be considered an apparent adsorption equilibrium constant. At $[L] = 1/K_L$, $[M_h] = [M_h\text{-}L_h]$, i.e., half of the surface site clusters have no ligand adsorbed while the other half are fully occupied by L. As each cluster has h sites, the concentration of free adsorption sites, [M], is given by

$$[\mathrm{M}] = h[\mathrm{M}_h] \qquad \text{Eq. S2}$$

Combining Eq. S1 and Eq. S2, we get

$$[\mathrm{M}_h\text{-}\mathrm{L}_h] = \frac{1}{h}[\mathrm{M}](K_\mathrm{L}[\mathrm{L}])^h \qquad \text{Eq. S3}$$

Second, considering the equilibrium of R adsorption (Langmuir adsorption, see Section 1.6) in the absence of the competing ligand:

$$\mathrm{M} + \mathrm{R} \leftrightarrow \mathrm{M\text{-}R}$$

K_R, the adsorption equilibrium constant of R, is

$$K_{\mathrm{R}} = \frac{[\text{M-R}]}{[\mathrm{M}][\mathrm{R}]} \quad \text{Eq. S4}$$

where [M-R] is the concentration of surface sites occupied by R.

In the case of R and L co-adsorption in which they do not interact other than competing for the surface adsorption sites, their respective adsorption equilibria still maintain as above. The equilibrium equations above are still valid. The total concentration of surface adsorption sites $[\mathrm{M}]_{\mathrm{T}}$ comprises three components:

$$[\mathrm{M}]_{\mathrm{T}} = [\mathrm{M}] + [\text{M-R}] + h[\mathrm{M_h}\text{-}\mathrm{L_h}] \quad \text{Eq. S5}$$

The coverage θ_{R} of R on the adsorption sites, by definition, is

$$\theta_{\mathrm{R}} = \frac{[\text{M-R}]}{[\mathrm{M}]_{\mathrm{T}}} \quad \text{Eq. S6}$$

Inserting Eq. S3 and Eq. S6 into Eq. S5, we get

$$\frac{[\text{M-R}]}{\theta_{\mathrm{R}}} = [\mathrm{M}] + [\text{M-R}] + [\mathrm{M}](K_{\mathrm{L}}[\mathrm{L}])^{h} \quad \text{Eq. S7}$$

After inserting Eq. S4 into Eq. S7 and rearranging, we get

$$\theta_{\mathrm{R}} = \frac{K_{\mathrm{R}}[\mathrm{R}]}{1 + K_{\mathrm{R}}[\mathrm{R}] + (K_{\mathrm{L}}[\mathrm{L}])^{h}} \quad \text{Eq. S8}$$

If N_{R} is the number of R molecules adsorbed on the surface sites of a catalyst particle, the reaction rate for the consumption of R on one particle is

$$v_{\mathrm{R}}\ (\mathrm{s}^{-1}\ \mathrm{particle}^{-1}) = k_{\mathrm{i}}k_{\mathrm{s}}N_{\mathrm{R}} \quad \text{Eq. S9}$$

Here k_{i} is the rate constant representing the intrinsic reactivity per site for the catalytic conversion; k_{s} is a scaling factor to describe the contribution of the co-reactant NH_2OH in the reaction and treated as a constant because NH_2OH is kept as a constant large excess in the experiments.

Let N_{T} be the total number of surface sites on one catalyst particle

$$N_{\mathrm{R}} = N_{\mathrm{T}}\theta_{\mathrm{R}} \quad \text{Eq. S10}$$

Therefore, combining Eq. S8, Eq. S9, and Eq. S10, we have

$$v_{\mathrm{R}} = k_i k_s N_T \theta_{\mathrm{R}} = k_{\mathrm{R}}\theta_{\mathrm{R}} = \frac{k_{\mathrm{R}}K_{\mathrm{R}}[\mathrm{R}]}{1 + K_{\mathrm{R}}[\mathrm{R}] + (K_{\mathrm{L}}[\mathrm{L}])^{h}} \quad \text{Eq. S11}$$

in which $k_{\mathrm{R}} = k_{\mathrm{i}}k_{\mathrm{s}}N_{\mathrm{T}}$ for brevity. Eq. S11 is give as Eq. 1 in the main text. When $h = 1$, i.e., no cooperative adsorption for ligand L, Eq. S11 becomes the case that both the reactant R and the competitor follow Langmuir adsorption, as the case in our initial development of COMPEITS imaging[22].

Note that the concept of a catalyst particle used in this derivation applies as well to an ensemble of many particles or a unit surface area, so the equations are also applicable to describe those cases.

Some other forms of Eq. S11 include:

$$v_{\mathrm{R}}^{-1} = \frac{1}{k_{\mathrm{R}}K_{\mathrm{R}}[\mathrm{R}]} + \frac{1}{k_{\mathrm{R}}} + \frac{(K_{\mathrm{L}}[\mathrm{L}])^{h}}{k_{\mathrm{R}}K_{\mathrm{R}}[\mathrm{R}]} \quad \text{Eq. S12}$$

Note in computing a COMPEITS image (e.g., Fig. 2a), $\Delta(n^{-1}) \propto \Delta v_{\mathrm{R}}^{-1} \propto (K_{\mathrm{L}}[\mathrm{L}])^{h} \propto (K_{\mathrm{L}})^{h}$, as in the following:

$$\Delta v_R^{-1} \equiv v_R^{-1}([L]) - v_R^{-1}([L]=0) = \frac{(K_L[L])^h}{k_R K_R [R]} \propto (K_L[L])^h \propto (K_L)^h \qquad \text{Eq. S13}$$

The Hill plot form of Eq. S11 is:

$$\log\left(\frac{k_R K_R [R]}{v_R} - K_R[R] - 1\right) \equiv Y = h \log [L] + h \log K_L \qquad \text{Eq. S14}$$

Here Y vs. log[L] is linear, and the slope is h, so-called Hill coefficient. *It is also worth noting that the Hill model of cooperativity was formulated specifically to treat positive cooperativity, where the Hill coefficient h (>1) corresponds to the minimum number of ligands that adsorb simultaneously* [19]*. If the cooperativity is negative, h is < 1 phenomenologically within the Hill model, but the physical interpretation of the value of h is undefined. For both cases, further deviation from h = 1 means larger cooperativity.*

1.6 Bulk measurements confirm the Langmuir-Hinshelwood kinetics of the fluorogenic auxiliary reaction, in which the reactant resazurin adsorbs noncooperatively

Bulk-level measurements of the fluorogenic auxiliary reaction kinetics (with and without ligand competition) not only confirm the validity of the COMPEITS approach on the sub-particle level, but also provide guidance on the choice of titration conditions for the single-molecule imaging experiments (see Methods).

The reaction rate v_R follows the Langmuir-Hinshelwood kinetics, as we showed earlier [23]:

$$v_R = \frac{k_R K_R [R]}{1 + K_R[R]} \qquad \text{Eq. S15}$$

k_R and K_R can be obtained via data fitting. If considering the cooperative adsorption of R, based on the Hill model, v_R would follow

$$v_R = \frac{k_R (K_R[R])^{h_R}}{1 + (K_R[R])^{h_R}} \qquad \text{Eq. S16}$$

Both Eq. S15 and Eq. S16 can fit the experimental v_R-[R] satisfactorily with equal quality (the two curves are overlapping with each other, Supplementary Fig. 3c). The fitted Hill coefficient h_R using Eq. S16 is 1 within experimental error, confirming that R does not adsorb cooperatively. Therefore, the Langmuir-Hinshelwood kinetics, i.e., Eq. S15, is sufficient for describing the catalytic kinetics of R to resorufin conversion, consistent with our previous work[23].

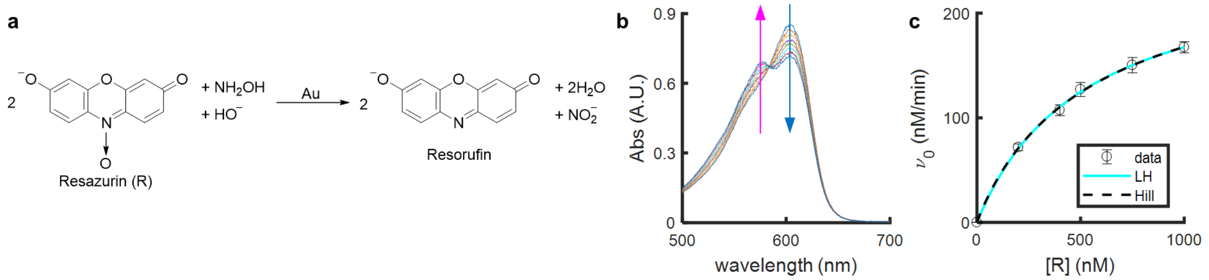

Supplementary Fig. 3 | Bulk measurements of 5-nm Au-nanoparticle catalyzed reduction of resazurin to resorufin by NH_2OH. a, Chemical equation of the fluorogenic auxiliary reaction. **b**, In situ absorption measurements of the reduction of R by NH_2OH catalyzed by Au nanoparticles in an aqueous solution. $[R]_0 = 4.0$ µM; $[NH_2OH]_0 = 1.0$ mM; [Au nanoparticle] = 0.010 nM (based on particles instead of atoms), in 7 mM pH 7.4 phosphate buffer. The blue and magenta arrows indicate the decrease of R peak at 602 nm and the increase of resorufin peak at 572 nm, respectively. **c**, The initial reaction rates vs. the R concentration. $[NH_2OH]_0 = 1.0$ mM; [Au nanoparticle] = 0.010 nM, in 7 mM pH 7.4 sodium phosphate buffer. Cyan solid line is the fits of Eq. S15 (the Langmuir-Hinshelwood model) with $k_R = 0.25 \pm 0.01$ µM/min, $K_{Rz} = 1.9 \pm 0.2$ µM^{-1}, the sum of squared residuals (SSR) is 18.5; the black dash line is the fit of Eq. S16 (the Hill model), with $k_R = 0.27 \pm 0.07$ µM/min, $K_{Rz} = 1.7 \pm 1.0$ µM^{-1}, $h_R = 0.96 \pm 0.23$, the SSR is 17.9. Error bars are s.d.

Next, the reaction rates were measured in the presence of different concentrations of ligands while keeping the concentration of R and everything else the same. In Eq. S11, [R] is known, and k_R and K_R obtained from Eq. S15 are used, while K_L and h (set as floating parameters) are obtained via fitting the v_R-[L] curve. Note that the value of K_R may vary due to the brand, amount, and freshness of 5-nm Au nanoparticles used, and it is the best practice to sonicate the stock solution before use to improve the quality and reproducibility of the results.

The results of bulk titration of ligands will appear in Section 2.

1.7 Single-molecule fluorescence imaging experiments

All single-molecule fluorescence microscopy experiments for COMPEITS imaging were carried out on a home-built prism-type wide-field total internal reflection fluorescence (TIRF) microscope (Olympus IX71, Supplementary Fig. 4a). A continuous wave circularly polarized 532 nm laser beam (CrystaLaser-GCL-025-L-0.5%) of ~10 mW was focused onto the sample (of ~60×100 μm^2) in a flow cell to directly excite the fluorescence of the catalytic product resorufin (Supplementary Fig. 4). A flow cell, 100 μm (height) × 5 cm (length) × 1 cm (width), formed by double-sided tape sandwiched between a quartz slide (Technical Glass) and a borosilicate coverslip (Gold Seal), was used to hold aqueous reactant solutions (and the competing ligand when applicable) for single-molecule fluorescence imaging measurements. The fluorescence emitted by the product was collected by a 60× NA1.2 water-immersion objective (UPLSAPO60XW, Olympus), filtered (HQ580m60, Chroma), and detected by a back-illuminated ANDOR iXon EMCCD camera (DU897D-CS0-#BV) operated at 30 ms frame rate for nanoplates or nanorods, and 100 ms per frame for 5-nm nanoparticles.

The 5-nm Au nanospheres, Au@mSiO_2 nanoplates or nanorods were dispersed on the quartz slide via drop-casting, dried, immobilized by heating in an oven at ~60 °C for 1-2 h, and then assembled into a microfluidic cell after UV-Ozone treatment. Reactant solutions were supplied into the flow cell at designed concentrations in a continuous flow at 10 $\mu L\ min^{-1}$ driven by a syringe pump (Chemyx incorporation). This flow-cell based reaction scheme provided a steady-state reaction condition, under which all single-molecule catalytic kinetics was measured.

Reductive deoxygenation of R to resorufin by NH_2OH (Supplementary Fig. 3a) was used as the fluorogenic auxiliary reaction for the COMPEITS imaging. Titration of R was performed for every sample considering the heterogeneity among single particles. All single-molecule imaging experiments were carried out at room temperature with 1 mM NH_2OH in 7 mM pH 7.4 phosphate buffer. The high NH_2OH concentration was to maintain a large excess so that NH_2OH is not a rate-limiting reagent in the catalysis (Supplementary Fig. 9m). Typically, 30,000 to 90,000 frames at 30 ms per frame were collected at one reactant concentration; 4 different reactant solutions with increasing concentrations of R from 0 to 0.3 μM were imaged, followed by 4 solutions with the highest concentration of R and increasing concentrations of a ligand. The concentrations of the ligands ranged from nM to mM, depending on the adsorption affinity of the ligands.

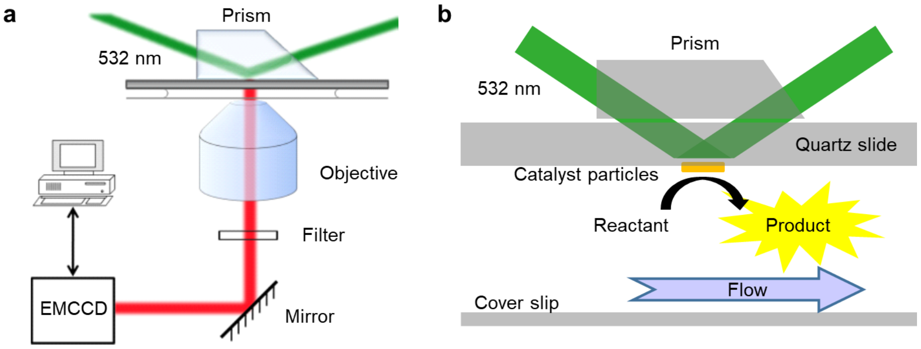

Supplementary Fig. 4 | The setups of imaging experiments. a, Schematics of prism-type TIRF microscope set up. Adapted with permission from ref[24]. **b**, Schematics of the flow reactor cell.

1.8 Single-molecule fluorescence image analysis

1.8.1 Single-molecule fluorescence image analysis for super-resolution localization

Identifying single fluorescent molecules

Information of single-molecule catalysis was extracted using a home-written MATLAB program from the fluorescence images in the movies, 'subtraction iQPALM' (image-based quantitative photo-activated localization microscopy, see Methods, Supplementary Codes). Briefly, each fluorescence image was first background subtracted to remove the constant emission from Au@mSiO_2 nanoplates or nanorods, where drift correction was also performed frame by frame so that the background was properly generated and subtracted. Such background subtraction is not needed for 5-nm Au nanoparticles. Afterwards, any pixel whose intensity value was greater than the mean pixel intensity plus 3~6 standard deviations was considered as a potential candidate product[22]. This intensity threshold typically yielded < 20 candidates per frame (each frame typically 60 × 100 μm^2). Usually a field of view (i.e., the image frame) from the optical microscope contained 20~50 particles, but only isolated individual particles with the targeted shapes (confirmed by SEM imaging) were selected for further analysis.

The centroid position (x_0, y_0) of each candidate product was determined by fitting a 13 × 13 pixel^2 area centered at the molecule's coordinate with a 2D Gaussian point spread function (PSF) (Eq. S17), where $I(x,y)$ is the EMCCD fluorescence intensity counts (*cts*) of the candidate at position (x, y) (nm); and A, B, and (σ_x, σ_y) are the amplitude, background, and standard deviations of the fitted 2D Gaussian function, respectively.

$$I(x,y) = Ae^{-\frac{1}{2}\left(\frac{x-x_0}{\sigma_x}\right)^2-\frac{1}{2}\left(\frac{y-y_0}{\sigma_y}\right)^2} + B \qquad \text{Eq. S17}$$

The values of σ_x and σ_y (Supplementary Fig. 5a-b) confirm the detection of single molecules (see 1.8.2 below)[25].

The total number of fluorescence photons (N) was obtained via Eq. S18, where g, S, and QE are the EM gain (unitless), sensitivity (electrons per count), and quantum yield (unitless) of the EMCCD camera in the spectral range of detected fluorescence, respectively. The constant 3.65 (eV per electron) accounts for electron creation in silicon, and E_{hv} (= 2.12 eV) is the energy of an individual fluorescence photon from the product molecule resorufin with an emission maximum wavelength at 585 nm.

$$N = 3.65\frac{(\text{cts}/g)(S/\text{QE})}{E_{\text{h}\upsilon}} \qquad \text{Eq. S18}$$

The localization error (Err_i, $i = x$ or y) of the centroid position was calculated as

$$\mathrm{Err}_i = \sqrt{\frac{\sigma_i^2}{N} + \frac{a_i^2}{12N} + \frac{8\pi\sigma_i^2 b^2}{a_i^2 N^2}} \qquad \text{Eq. S19}$$

where a is the pixel size, and b is the standard deviation of the spatially non-uniform image background[7,26,27]. The one-dimensional localization error is typically ~27 nm at 30 ms frame rate for imaging reactions on Au nanoplates (Supplementary Fig. 5d-e) and can be ~10 nm at 100 ms frame rate for imaging reactions on 5-nm Au nanoparticles. The symbol N in Eq. S18 and Eq. S19 represents the number of photons impinging on the camera and the number of photons detected, respectively. However, the ratio of these two numbers is the quantum yield, i.e., QE in Eq. S18, which has a value of 95-97% for our camera in the fluorescence detection spectral region (550 – 610 nm). The difference in N is only ~3-5%, and the effect on the localization error Err_i is even smaller and negligible.

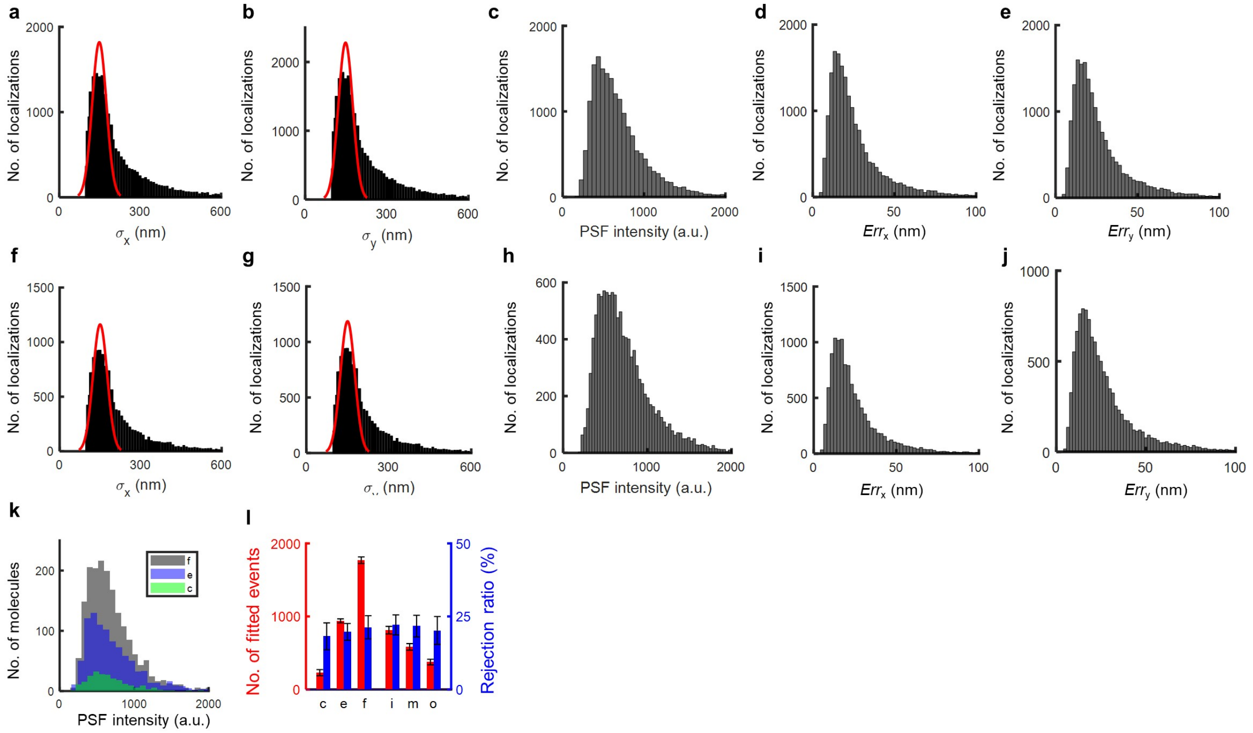

Supplementary Fig. 5 | Parameters and localization errors of single-molecule fluorescence image analysis. a-e, Distributions of σ_x (**a**), σ_y (**b**), PSF intensity (i.e., the volume of the fitted 2D Gaussian function) (**c**), Err_x (**d**), and Err_y (**e**) from a Au nanoplate in a typical imaging experiment. Red lines are Gaussian fits for the bins in 100-200 nm range in **a-b**, and the fitted averages are 148 ± 26 nm and 149 ± 26 nm, respectively. The averages of **c-e** are 690 ± 342, 26 ± 18 nm, and 27 ± 19 nm, respectively. Errors here are s.d. **f-j**, Distributions of parameter as in **a-e** using a pixel-integrated PSF: $I(x,y) = A + Bx + Cy + \int_{x-\delta}^{x+\delta} dX \int_{y-\delta}^{y+\delta} dY I_0 \exp\left[-\frac{1}{2}\left(\frac{X-x_0}{\sigma_x}\right)^2 - \frac{1}{2}\left(\frac{Y-y_0}{\sigma_y}\right)^2\right]$, where $I(x,y)$ is the intensity counts of the fluorescent molecule in the image at position (x,y), $A+Bx+Cy$ is a sloping plane to account for the background in the fitting, $I_0 \exp\left[-\frac{1}{2}\left(\frac{X-x_0}{\sigma_x}\right)^2 - \frac{1}{2}\left(\frac{Y-y_0}{\sigma_y}\right)^2\right]$ is a two-dimensional Gaussian function, and δ is half of the pixel size. Along x or y axis, the integration over each pixel is done numerically by dividing each pixel into 11 equal segments. (x_0, y_0) gives the center location of the PSF (see details in Ref[7,28,29]) instead of Eq. S17: σ_x (**f**), σ_y (**g**), PSF intensity (i.e., the volume of the fitted PSF) (**h**), Err_x (**i**), and Err_y (**j**) from a Au nanoplate in a typical imaging experiment. Red lines are Gaussian fits for the bins in 100-200 nm range in **f-g**, and the fitted averages are 150 ± 26 nm and 151 ± 26 nm, respectively. The averages of **h-j** are 734 ± 346, 25 ± 17 nm, and 27 ± 19 nm, respectively. Errors here are s.d. The results in **f-j** are similar to those in **a-e**, indicating that the two different PSF forms give essentially the same results. **k**, Histograms of PSF intensities of product molecules detected in the corner, edge, and flat facet regions, and the averages and s.d. are 678 ± 308 at the corner region, 710 ± 354 at the edge region, and 689 ± 323 at the flat facet region. The PSF intensities are essentially the same across the different regions, indicating there is no spatial bias in the detection of the products of the fluorogenic auxiliary reaction. **l**, The number of fitted localizations and the overall rejection rates at different

regions of nanoplates. Rejections consist of filtering based on σ_x and σ_y (either too small or too big) and diffusing molecules (see Section 1.8.2). The data show that there is no significant difference in the rejection rate across different regions. Data were averaged from 55 nanoplates, [R] = 0.2 μM, [NH_2OH] = 1.0 mM, and no ligand. Error bars are s.e.m.

<u>Correction for the drift of the stage</u>

Each set of titration experiments lasted for a few hours, during which the microscope stage and the flow cell could drift by hundreds of nanometers. The sample drifting within the same movie, which affected the localization of centroid positions of the candidate product molecules, was corrected for (see Supplementary Codes). Both Au nanoplates and nanorods show stable intrinsic emission under 532 nm irradiation and their positions can be determined by the PSF fitting of their emission, so they can act as internal position markers in the frames; as the catalysis events are sparse, any contribution of the product fluorescence on top of a nanoplate/nanorod in a small fraction of image frames is washed out by averaging over multiple nanoplate/nanorod position markers. The microscope stage drift was monitored in a frame-by-frame fashion by calculating the intensity-weighted centroid position of the stable intrinsic photoluminescence of the Au nanoplates and nanorods. The average drift of multiple particles (>5) present in the same movie was used to correct the centroid position of each candidate fluorescent product molecule. Because the Au nanoplates and nanorods are constantly bright objects, prior to extraction of single-molecule fluorescence signals from the images, the average emission of Au particles was subtracted. The last 1000 frames of a movie were averaged to generate the average emission image. Each frame in the catalysis experiment in the presence of fluorogenic reactant was subtracted by the stage-drift-corrected, averaged emission image. Before the subtraction of the Au nanoplates and nanorods emissions, images to be subtracted are expanded by 10 times in x and y dimensions using the bilinear interpolation method to accommodate corrections that are subpixel-level stage drifts and, after subtraction, the expanded images are re-binned to the original image size (i.e., decreasing the image size by 10 times in both x and y dimensions).

The 5-nm Au nanoparticles are not emissive under 532 nm irradiation, so additional 100-nm Au nanoparticles that are emissive were added as position markers into the corresponding flow cells. The positions of at least 5 position markers were averaged for the drift correction in a frame-by-frame manner.

1.8.2 Quantitative single-molecule counting algorithm to correct for over-counting and underestimation of product molecules

The raw candidate product molecules with PSF fitting parameters were filtered by a quantitative single-molecule counting algorithm, to remove spurious detections and noise contributions, and correct for unresolved multiple-molecule detections, as well as over-counting due to a product molecule adsorbed on the nanoparticle for multiple frames (i.e., multi-frame events). A flow chart of the algorithm is given in our previous study[26]. Briefly, first, the diffraction-limited width of a single-molecule PSF is $0.61\times\lambda/\mathrm{NA} = 0.61\times585\ \mathrm{nm}/1.2 = 297\ \mathrm{nm}$ (NA is numerical aperture), corresponding to $\sigma_{x/y} \approx 297/2.355 = 126$ nm, so candidate events with their PSF σ_x or σ_y below 100 nm (coming from 'hot pixels') are excluded. Next, candidate product molecules with a PSF width ranging from 100 nm and to a threshold value (σ_{thres}, set as the smaller value of mean σ_x or σ_y plus 2 standard deviations, usually ~220 nm) are selected as single-molecule events.

For those candidate events with their PSF $\sigma_{x/y}$ greater than σ_{thres}, if their PSF intensity is greater than the PSF intensity of a single-molecule event, they were treated as multiple-molecule events, where the number of molecules was determined by the PSF intensity of the event divided by that of a single-molecule event and rounded to the nearest integer. Otherwise (i.e., the PSF intensity is less than that of a single-molecule event), they were treated as molecules that diffused significantly on the catalyst surface (about 5% of the observed events) and excluded from further analysis.

In addition, for two molecules detected in two consecutive frames and the distance between their centroid locations in these two frames is less than $2\times\mathrm{Err}_{\mathrm{OM}}$ ($\mathrm{Err}_{\mathrm{OM}} = \sqrt{\mathrm{Err}_x^{\,2} + \mathrm{Err}_y^{\,2}}$, ~40 nm, see

Supplementary Fig. 5d-e), these two molecules were considered as one product molecule adsorbed on the catalyst particle for a time longer than a single frame acquisition time, i.e., a multi-frame event. Thus, only the position in the first frame was kept and counted only once to not overestimate the catalytic activity due to such multi-frame events (less than 1% of the observed events).

Both single-molecule and multiple-molecule events were counted for the calculation of specific reaction rates (see Section 1.8.4).

1.8.3 Overlay of SEM and optical microscopy (OM) images

Before analyzing the COMPEITS titration results of single nanoplates or nanorods, the positions of the fluorescent catalytic events on each nanoplate or nanorod were mapped onto its structure extracted from its SEM image, utilizing a bright field optical microscopy (OM) image (transmission mode) that shares the same coordinate system with the fluorescence images (see Supplementary Codes). The procedure is similar to our previous work[2] and briefly summarized in Supplementary Fig. 6, with the estimation of errors listed in Supplementary Table 1. We did not perform this overlay procedure for the 5-nm Au nanoparticles as they are smaller than the ~40 nm overall error.

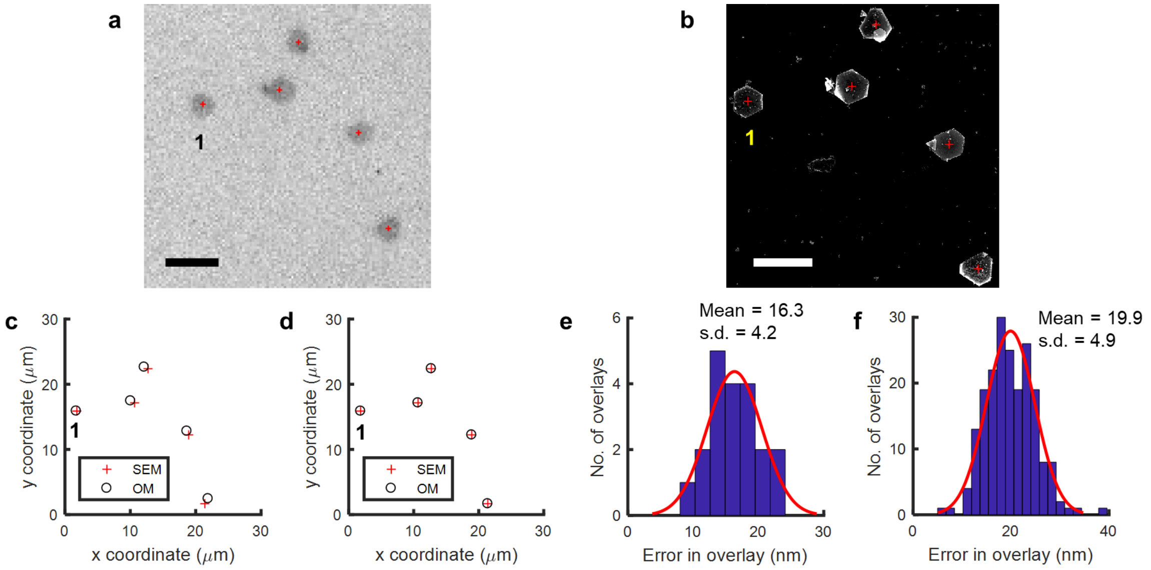

Supplementary Fig. 6 | Representative procedure of overlaying the centroid positions of Au nanoplates visible in a bright field optical transmission image and in an SEM image. a, Optical microscopy (OM) image showing the centroid positions of the Au nanoplates marked by red crosses, detected from an edge-detection algorithm. **b**, SEM image corresponding to the same sample area, also with red crosses showing the centroid positions of the nanoplates, determined from a similar edge-detection algorithm. The scale bars are 5 μm in **a** and **b**. **c**, The coordinates of the centroid positions of nanoplates determined from OM as well as from SEM, after translating the OM coordinates of Particle 1 to overlap with the SEM counterpart. **d**, The same SEM coordinates as in (**c**), along with the OM coordinates after expansion and rotational operations using Particle 1 as the reference position. The expansion and rotational matrix are determined by the average of all pairwise changes in distance and angle. **e**, Histogram of the overlay errors from the example sample area shown in **a**-**d**. **f**, Histogram of the overlay errors from all nanoplates analyzed in a flow cell.

Supplementary Table 1 | Estimations of localization errors (using data from nanoplates as examples).

Error source	Super-resolution fluorescence imaging (ε_1)	SEM imaging (ε_2)	OM-SEM overlay errors (ε_3)	Overall errors (ε) $\varepsilon = (\varepsilon_1^2 + \varepsilon_2^2 + \varepsilon_3^2)^{1/2}$
Estimated error (nm)	27	20	20	39

1.8.4 Obtaining v_R for a whole particle or different sub-particle sections

<u>Obtaining v_R for 5-nm Au nanoparticles</u>

Since the 5-nm Au nanoparticles were not emissive under 532 nm light illumination, they were identified as small areas (e.g., 40 × 40 nm^2) with recurring fluorescent bursts that report catalytic reactions as in our previous work[23]. The reaction rate v_R of a single nanoparticle (in s^{-1} particle^{-1}) was calculated from the number of product molecules on a particle divided by the corresponding reaction time.

<u>Obtaining v_R for Au nanoplates at the sub-particle level</u>

Each nanoplate was dissected into different sections according to its SEM image and the geometric relations outlined below, and the product molecules were grouped into sections based on their positions after transformation onto the same coordinate system of the SEM image of the nanoplate (see Supplementary Codes).

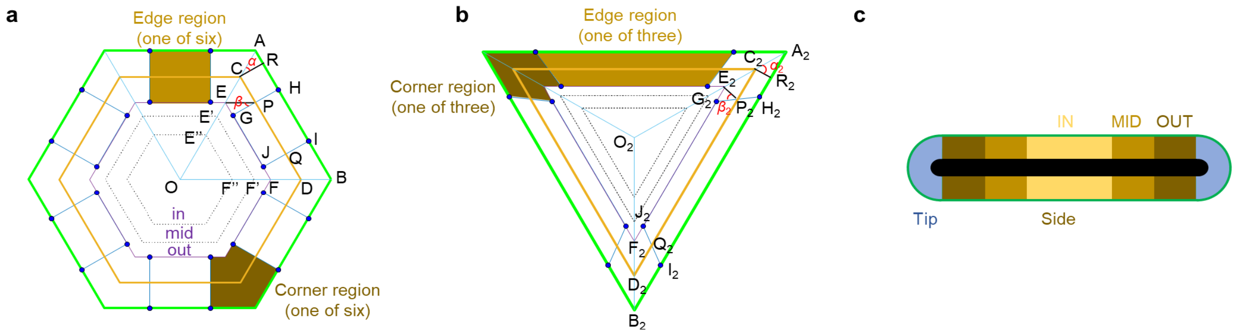

Supplementary Fig. 7 | Schematics of dissecting single hexagonal nanoplates (a), triangular nanoplates (b), and nanorods (c). Relative sizes of each region are not drawn to scale and the exact sizes may differ from one particle to another.

Supplementary Fig. 7a shows the schematics of how each nanoplate is dissected into different sections. The outer edge of the mesoporous silica shell, e.g., AB (green), and the contour of the Au core, e.g., CD (golden), are directly visible from the SEM image (Supplementary Fig. 1c, Supplementary Fig. 6b). Therefore, the coordinates of vertices such as A, B, C, and D are obtained from the edge detection algorithm for each nanoplate. Let CR be perpendicular to AB; then the length of CR, which is the thickness (t) of the $mSiO_2$ shell, can be measured for individual nanoplates. Point O is the geometric center, and E and F are points on OC and OD, respectively, where CE = DF ≡ 3ε, where ε is the overall localization error of the correlated SEM-fluorescence imaging method. The value of ε is about 40 nm in this work (see Supplementary Table 1). Therefore, the area enclosed by the purple lines including EF define the flat facet region. Points P and Q are on CD, where CP = DQ = 3ε. GH and IJ are perpendicular to CD, and GHIJ (such as the area highlighted in dark yellow) defines one of the edge regions. Regions outside the flat facet and the edge regions are the corner regions (such as the area highlighted in dark brown). The boundaries of the corner, edge, and flat facet regions, which are needed to sort product molecules and for the calculation of surface area, can be expressed based on these known coordinates in addition to ε in the following way.

From $\overrightarrow{\mathrm{CE}} = \frac{|\mathrm{CE}|}{|\mathrm{CO}|}\overrightarrow{\mathrm{CO}}$, $x_\mathrm{E} = \frac{3\varepsilon}{|\mathrm{CO}|}(x_\mathrm{O} - x_\mathrm{C}) + x_\mathrm{C}$, in which all quantities are known. Similarly, y_E, x_F, and y_F can be solved. We also get $x_\mathrm{H} = \frac{|\mathrm{AH}|}{|\mathrm{AB}|}(x_\mathrm{B} - x_\mathrm{A}) + x_\mathrm{A}$, $x_\mathrm{I} = \frac{|\mathrm{AH}|}{|\mathrm{AB}|}(x_\mathrm{A} - x_\mathrm{B}) + x_\mathrm{B}$ (note |AH| = |IB|), $x_\mathrm{J} = \frac{|\mathrm{EG}|}{|\mathrm{EF}|}(x_\mathrm{E} - x_\mathrm{F}) + x_\mathrm{F}$ (note |EG| = |JF|), $x_\mathrm{G} = \frac{|\mathrm{EG}|}{|\mathrm{EF}|}(x_\mathrm{F} - x_\mathrm{E}) + x_\mathrm{E}$, where |AH| = |AR|+|RH| = |AC|×sin(α) + 3ε; |EG| = |EP|×sin(β). All the y coordinates can be expressed in the y counterparts.

For equilateral hexagons, $\alpha = \beta = \pi/6$, |EP| = |CE| = 3ε; and these values are used for all other nanoplates as an approximation. The extreme variation from a hexagonal shape for the nanoplates is the equilateral triangular shape. The coordinates of boundaries established on the equilateral hexagon (i.e., α = β = π/6) can also dissect an equilateral triangle into the corner regions, edge regions, and the flat facet

reasonably well (Supplementary Fig. 7b), although the corresponding values of α_2 and β_2 differ from π/6. In this case, G_2H_2 and I_2J_2 are no longer perpendicular to C_2D_2, but $G_2H_2I_2J_2$ (such as the area highlighted in dark yellow) is still a satisfactory representation of the edge region. Therefore, it is practicable to apply these definitions of nanoplate dissection to all nanoplates.

The flat facet region of each nanoplate is further divided into three sections, i.e., inner, middle, outer, with equal surface areas, separated in dotted lines (e.g., E''F'' and E'F', where |OF''|:|OF'|:|OF| = $\sqrt{1/3}$: $\sqrt{2/3}$:1). Specific activities in each section were obtained accordingly.

After assigning the product molecules into different regions, ν_R (in s^{-1} μm^{-2}) was calculated as the number of product molecules divided by reaction time, then divided by the surface area of the region. The surface area used in these calculations were of the 3D nanoplates instead of the 2D projection. To be specific, both the top and bottom areas were counted for the flat facet, and the edge region consisted of the side plane (approximated to be vertical to the basal plane, not seen in the 2D projection) and the parts from the top or bottom basal planes. Note the bottom side of the flat facets faces the supporting quartz slide; the inclusion of this bottom side or not in calculating the catalytic surface area only affects the absolute value of the measured specific turnover rate, but does not affect its dependences on the concentrations of the reactant or the competitor and therefore does not affect the value of determined adsorption equilibrium constants.

Obtaining ν_R for Au nanorods at the sub-particle level

The structural contour of each nanorod was first estimated in SEM image from the edge detection function, then fitted by a rectangle fused with two semicircles at the two short sides (Supplementary Fig. 7b, see Supplementary Codes)[7]. The rectangle was defined as the side region, while the two semicircles were defined as the tip regions. The side region is further divided into three sections with equal areas, i.e., IN, MID, OUT. Similarly, ν_R (in s^{-1} μm^{-2}) was calculated as the number of product molecules divided by reaction time, then divided by the surface area of the region. The surface area also considered the 3D geometry, i.e., two hemisphere for the tip region and cylinder for the side region.

1.9 Facet-controlled synthesis of colloidal Au nanoparticles in the presence of increasing [CTAB] and their SEM characterization: transition from irregular-shaped nanoparticles to high-quality nanoplates

Au nanoparticles were synthesized via reduction of $HAuCl_4$ by ascorbic acid in the presence of various [CTAB] in aqueous solution, modified from an earlier protocol[30] (see Methods). Supplementary Fig. 8 shows the larger-area SEM images of Au nanoparticles synthesized at increasing [CTAB], indicating gradual progression from irregular-shaped nanoparticles to a mixture of irregular nanoparticles and nanoplates and further to high-quality nanoplates; the corresponding CVs of Pb UPD are described in the main text (Fig. 4d-i).

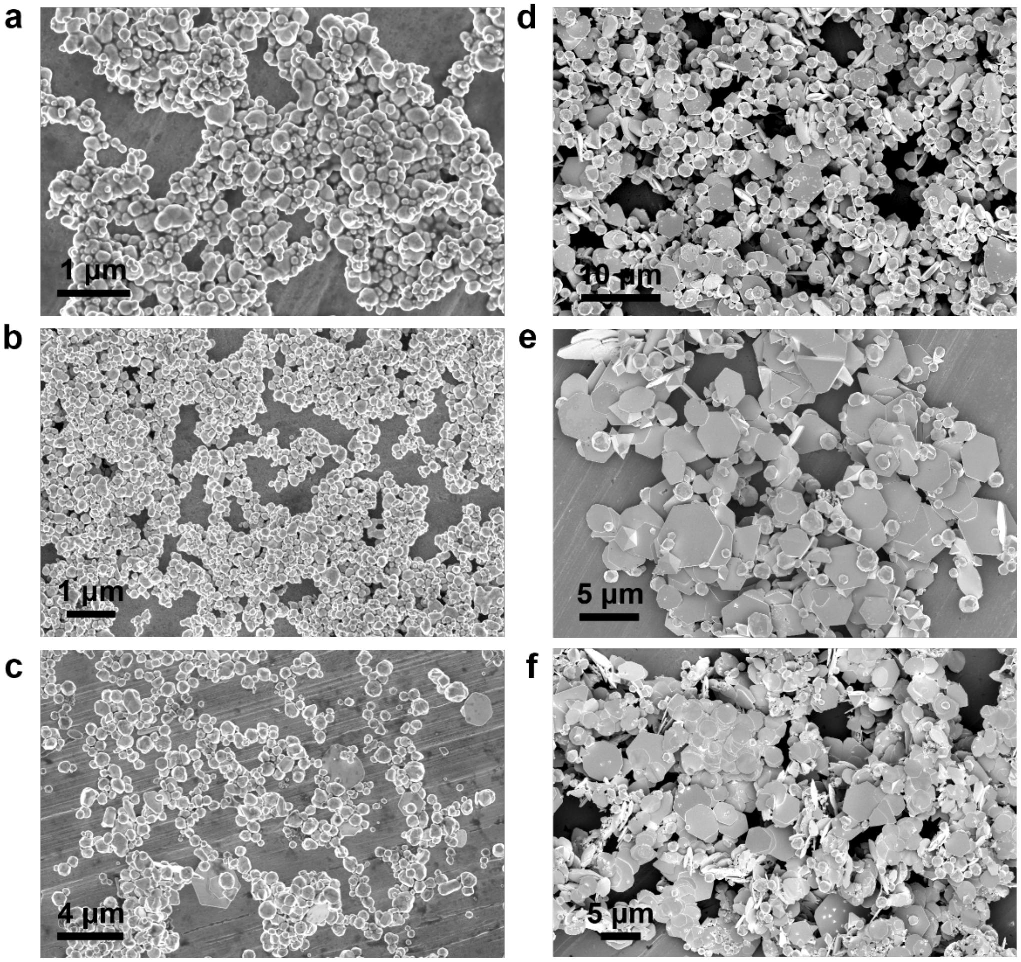

Supplementary Fig. 8 | SEM images showing a large area of the Au nanoparticles synthesized at various concentrations of CTAB: 0.26 mM (**a**), 0.52 mM (**b**), 1.04 mM (**c**), 1.56 mM (**d**), 2.34 mM (**e**), and 3.12 mM (**f**) (Supplementary Information section 1.9). The transition from irregular nanoparticles to nanoplates supports the increasing proportion of {111} facets on the particle surfaces in the samples.

2 Supplementary bulk reaction titration confirms: (1) CTAB/CTAOH/CTAC adsorb with positive cooperativity; (2) PVPs adsorb with negative cooperativity; (3) I^-/Br^-/BME adsorb non-cooperatively; (4) monomeric VP, EtOH, and K^+ have negligible adsorption; (5) [NH_2OH] is saturated for the fluorogenic reaction kinetics

The titration results summarized in Supplementary Fig. 9 and Supplementary Table 2a confirmed that many ligands (Supplementary Fig. 9a-i) could indeed suppress the reaction rate of 5-nm Au-nanoparticle catalyzed reduction of resazurin to resorufin by NH_2OH, while some could not (Supplementary Fig. 9j-l). Among the ligands studied, CTAB, CTAOH, and CTAC showed positive adsorption cooperativity, PVP of different molecular weights showed negative cooperativity, and BME, I^-, and Br^- showed no cooperativity. These results laid the foundations of single-molecule experiments.

Titration of VP (Supplementary Fig. 9j) and EtOH (Supplementary Fig. 9k) showed no apparent suppression of the fluorogenic reaction rate, indicating that they do not have significant adsorption on Au nanoparticles in the concentration range studied under the measurement conditions.

To ensure the slightly basic pH condition during the titration, 7 mM phosphate (mixture of K_2HPO_4 and KH_2PO_4) was used to maintain pH 7.4. Changing the phosphate buffer concentration from 7 mM to 3.5 mM or 14 mM did not change the rate of the fluorogenic reaction rate v_R without or with ligands

(Supplementary Fig. 9l). Therefore, millimolar potassium and phosphate ions show negligible adsorption on the Au particles. The adsorption of KI or KBr is attributable to I^-, or Br^-, where the adsorption of potassium is minimal.

Control experiments under the COMPEITS conditions and under various [NH_2OH] (in the presence of CTAB, Supplementary Fig. 9m) show that the reaction rate stays unchanged, which confirms that the concentration of NH_2OH was indeed in excess (i.e., kinetically saturated) and CTAB-adsorption-induced suppression of the fluorogenic reaction rate is due to competition with the reactant resazurin not the co-reactant NH_2OH.

The different values of K_R measured across different ligands could be attributed to different conditions of the nanoparticles used (i.e., different batches, freshness, extent of mixing in the cuvette), while k_R is further affected by the amount of nanoparticles used.

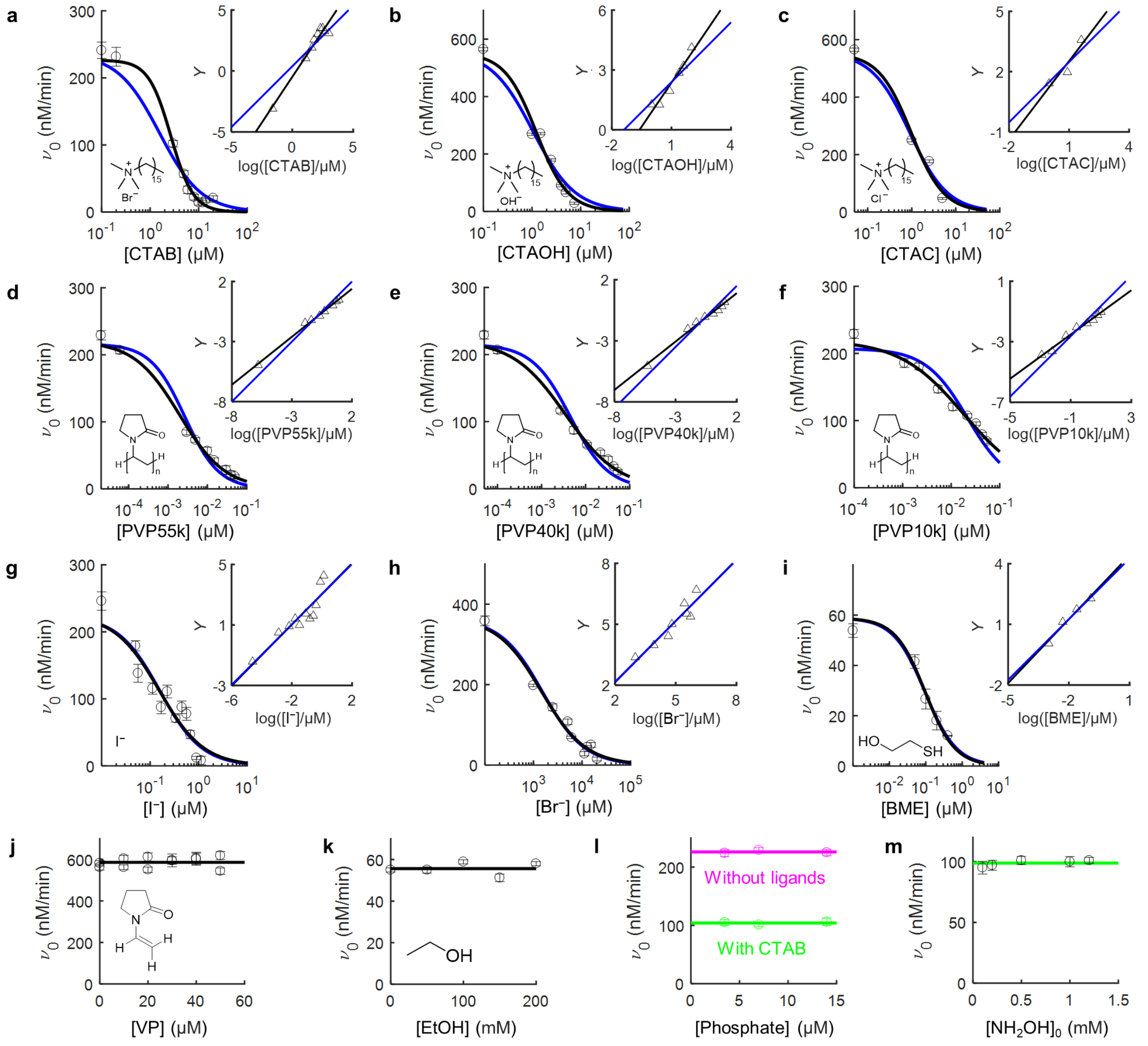

Supplementary Fig. 9 | Bulk initial reaction rate v_0 of 5-nm Au-nanoparticle catalyzed reduction of resazurin to resorufin by NH_2OH in the presence of competing ligands at different concentrations [L] (Supplementary Information sections 2)**. a-k,** Ligands CTAB (**a**), CTAOH (**b**), CTAC (**c**), PVP55k (**d**), PVP40k (**e**), PVP10k (**f**), I^- (**g**), Br^- (**h**), BME (**i**), VP (**j**), and ethanol (**k**). Data points at [L] = 0 are placed on the *y*-axes manually. Typically, $[R]_0 = 1.0 \sim 10$ μM; $[NH_2OH]_0 = 1.0$ mM; [Au nanoparticle]

= 0.010 ~ 0.10 nM (based on particles instead of atoms), in 7 mM pH 7.4 phosphate buffer, and the conditions for different points within a panel is the same except the ligand concentration. Black lines: fits with Eq. S11; blue lines: fits with Eq. S11 where h is fixed to 1; insets: the corresponding Hill plots following Eq. S14. The fitting parameters of **a**-**i** are summarized in Supplementary Table 2a. **l**, Control experiments at different [phosphate]. **m**, Control experiments with various $[NH_2OH]_0$ in the presence of 3 μM CTAB as one point in **a**. Solid line in **l**-**m**: the average v_0 to guide the eye.

Supplementary Table 2 | Summary of fitting parameters of bulk titration curves of 5-nm Au nanoparticles shown in Supplementary Fig. 9 (a) and summary of parameters extracted from single 5-nm Au nanoparticle titration curves shown in Supplementary Fig. 12(b). Errors in **a** are s.d. estimated from fitting; values in **b** are the mean and s.e.m. of the fitting parameters from all the nanoparticles analyzed. Note that the values in **b** are the averages of fitting results of individual particles, so they can be different from values of the black curve in Fig. 1e, which are fits of the average rate.

a. Fitting parameters of bulk ligand competition titration curves on 5-nm Au nanoparticles (Supplementary Information section 2)

Ligand	K_L (M^{-1})	h	K_R (μM^{-1})	k_R ($\mu M\ min^{-1}$)
CTAB	6.0 ± 1.2 (×10^5)	1.8 ± 0.4	1.3 ± 0.7	0.49 ± 0.15
CTAOH	2.2 ± 0.7 (×10^6)	1.3 ± 0.2	6.1 ± 1.7	0.77 ± 0.07
CTAC	3.5 ± 1.1 (×10^6)	1.2 ± 0.1	6.0 ± 1.0	0.78 ± 0.09
PVP55k	2.5 ± 0.8 (×10^9)	0.81 ± 0.11	2.4 ± 0.6	0.34 ± 0.04
PVP40k	1.6 ± 0.8 (×10^9)	0.71 ± 0.09	2.4 ± 0.6	0.34 ± 0.03
PVP10k	4.2 ± 1.4 (×10^8)	0.63 ± 0.07	2.5 ± 0.6	0.34 ± 0.03
I^- [a]	2.1 ± 0.8 (×10^7)	0.9 ± 0.2	2.1 ± 1.0	0.37 ± 0.08
I^- (h = 1)	1.8 ± 0.5 (×10^7)	1	2.4 ± 1.1	0.34 ± 0.07
Br^- [a]	1.6 ± 0.6 (×10^3)	1.0 ± 0.2	1.1 ± 0.6	0.80 ± 0.25
Br^- (h = 1)	1.5 ± 0.3 (×10^3)	1	1.1 ± 0.5	0.78 ± 0.23
BME [a]	3.2 ± 0.7 (×10^7)	1.1 ± 0.2	2.1 ± 0.5	0.10 ± 0.01
BME (h = 1)	3.5 ± 0.5 (×10^7)	1	2.1 ± 0.5	0.10 ± 0.01
VP	n.d. [b]	n.d. [b]	n.d. [b]	n.d. [b]
EtOH	n.d. [b]	n.d. [b]	n.d. [b]	n.d. [b]

b. Fitting parameters of single 5-nm Au-nanoparticle COMPEITS titrations averaged over many particles (Supplementary Information section 3.4)

Ligand	No. of nanoparticles	K_L (M^{-1})	h	K_R (μM^{-1})	k_R (s^{-1} particle^{-1})
CTAB	50	6.6 ± 0.2 (×10^5)	2.0 ± 0.1	7.6 ± 0.4	0.39 ± 0.08
CTAOH	43	2.2 ± 0.1 (×10^6)	1.3 ± 0.1	7.9 ± 0.3	0.37 ± 0.04
CTAC	47	4.7 ± 0.1 (×10^6)	1.1 ± 0.1	7.7 ± 0.2	0.37 ± 0.03
PVP55k	36	2.0 ± 0.2 (×10^9)	0.75 ± 0.03	8.3 ± 0.3	0.36 ± 0.04
PVP40k	42	1.4 ± 0.1 (×10^9)	0.68 ± 0.02	8.0 ± 0.2	0.36 ± 0.03
PVP10k	33	2.9 ± 0.3 (×10^8)	0.64 ± 0.02	8.5 ± 0.3	0.35 ± 0.04
I^- [a]	44	2.1 ± 0.8 (×10^7)	0.9 ± 0.2	8.7 ± 0.4	0.36 ± 0.05
I^- (h = 1)	44	1.8 ± 0.5 (×10^7)	1	8.0 ± 0.3	0.37 ± 0.05
Br^- [a]	39	1.3 ± 0.6 (×10^3)	1.0 ± 0.1	8.0 ± 0.4	0.38 ± 0.07
Br^- (h = 1)	39	1.2 ± 0.3 (×10^3)	1	7.8 ± 0.4	0.38 ± 0.06
BME [a]	40	2.4 ± 0.1 (×10^7)	1.0 ± 0.1	8.2 ± 0.3	0.36 ± 0.03
BME (h = 1)	40	2.6 ± 0.1 (×10^7)	1	8.1 ± 0.3	0.36 ± 0.03

[a] The fitted h values of these ligands were equal to 1 within error, so the h was then fixed to 1 for these ligands to obtain K_L more accurately. [b] n.d., not determined.

3 Supplementary results of single-molecule reaction imaging and COMPEITS imaging of single 5-nm Au nanoparticles

3.1 Super-resolution images of fluorogenic auxiliary reaction and COMPEITS images of ligand adsorption

Motivated by the bulk experiments, we moved on to single-molecule imaging of catalytic reactions on 5-nm Au nanoparticles first, with concentration titrations of the fluorogenic reactant and the competing ligands. We optimized the amount of 5-nm Au nanoparticles to be drop casted onto the quartz slide of the flow cell to have low density and ensure minimal clustering of particles. For rare occurrences of particle

clustering that are not resolvable at ~10 nm resolution, the measured affinity and cooperativity are the averages of the clustered particles.

Supplementary Fig. 10a shows a segment of a typical fluorescence trajectory of a single 5-nm Au nanoparticle catalyzing the fluorogenic auxiliary reduction reaction of resazurin; each fluorescent burst represents the formation of one product molecule resorufin and its subsequent desorption from the nanoparticle surface. In the absence of CTAB, the localized positions of individual reaction products on a single 5-nm particle span a region of ~10 nm in size (Supplementary Fig. 10b), the effective spatial resolution of our imaging technique — note this resolution depends on the localization accuracy of individual molecules (see Eq. S19), which in turn depends on the S/N ratio of fluorescence detection and thus on the particular imaging experiment of different catalyst particles; the typically resolution is about 10-40 nm. Upon adding CTAB, the number of detected products (n) of the fluorogenic auxiliary reaction decreases, reflecting CTAB competition with resazurin adsorption on the particle (Supplementary Fig. 10c).

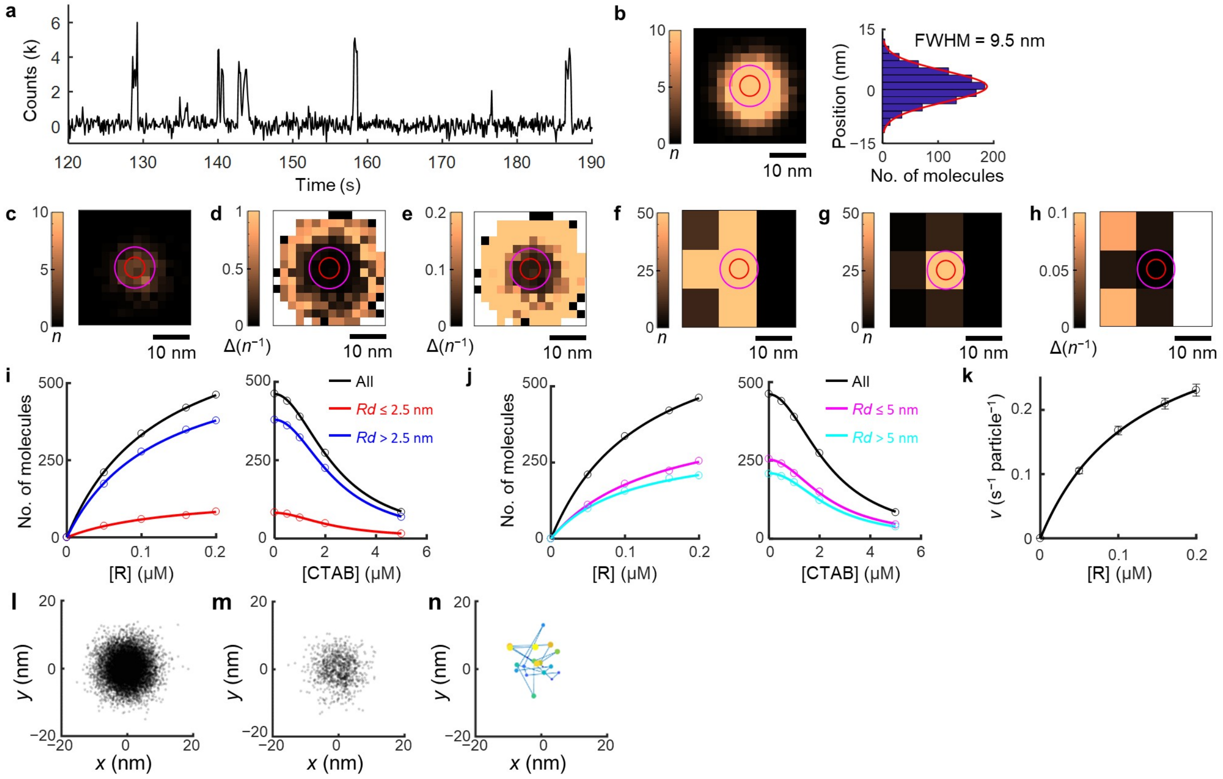

Supplementary Fig. 10 | COMPEITS images and titrations of ligand adsorption on single 5-nm Au nanoparticles. a, A segment of an exemplary fluorescence intensity vs. time trajectory on a single 5-nm Au nanoparticle. **b-c**, 2D histograms of the fluorogenic auxiliary reaction product molecules on a single 5-nm Au nanoparticle detected over 90 min without (**b**) and with 5 μM CTAB (**c**). [R] = 200 nM, [NH_2OH] = 1 mM. Pixel size: 2 × 2 nm^2. All scale bars are 10 nm. Right in **b**: 1D histogram in the y direction; FWHM is 9.5 nm, showing the spatial resolution here is ~10 nm. **d**, The COMPEITS image derived from (**b**) and (**c**). n: number of fluorogenic probe reaction products detected over 90 min. $\Delta(n^{-1}) \propto \Delta(v^{-1}) \propto K_L^h$ based on Eq. (1) and Eq. S13. **e**, Same as **d**, with the color scale adjusted to focus on smaller values. Red line: estimated structural contour of the 5-nm Au nanoparticle, where the center is estimated from the center of Gaussian fittings of the spatial distribution of product molecules. Magenta line: a circle with a diameter of 10 nm, the rounded full-width-at-half-maximum of the Gaussian distribution of product molecules. **f-h**, Same images corresponding to **b-d**, with pixel size 10 × 10 nm^2. COMPEITS images: white/null pixels represent occasional negative values or infinities due to 1/0 calculations. **i**, The titration of product molecules with different concentrations of R (left) and CTAB (right) within the whole area (black), the subset within the contour of the 5-nm Au nanoparticle (red) and the subset outside (blue). The fitted K_{CTAB} are 0.68 ± 0.01, 0.68 ± 0.05, and 0.68 ± 0.02 μM^{-1}, respectively; and the fitted h are 2.0 ± 0.1, 1.9 ± 0.2, and 2.0 ± 0.1, respectively. **j**, Similar to **i**, but separating the subset within the contour of a circle of 10 nm diameter (magenta) and the subset outside (cyan). The fitted K_{CTAB} are 0.66 ± 0.03 and 0.70 ± 0.03 μM^{-1}, respectively; and the fitted h are 1.9 ± 0.1 and 2.0 ± 0.1, respectively. Rd is the radius of the red (**i**) or the magenta (**j**) circle. **k**, Single-particle titration of auxiliary reaction rate v_R vs. [R] averaged over 50 particles, each of which was later titrated in [CTAB] as shown in Fig. 1b. Black line: fits

with Eq. S15, where $k_R = 0.39 \pm 0.08$ s^{-1} $particle^{-1}$, $K_R = 7.6 \pm 0.4$ μM^{-1} (also in Supplementary Table 2b). **l**-**m**, Scatter plots of molecular localizations corresponding to **b**-**c**. **n**, Distribution of localizations for a product molecule lasting for 19 frames on a 5-nm Au nanoparticle, with the size of the symbol marker increasing with the frame index.

A COMPEITS image is generated by the inverse subtraction of two super-resolution fluorescence localization images, where the value of each pixel is calculated as $\Delta(n^{-1}) = 1/n_2 - 1/n_1$, where the subscript 1 and 2 represents the images with zero and a certain [L] (>0), respectively. Note the pixels of each of these two original image (n_1 or n_2) store the counts of fluorescent products generated in the corresponding space over the same period of time, e.g., 15 min. As $\Delta(n^{-1}) \propto \Delta(v^{-1}) \propto K_L^h$ based on Eq. (1) and Eq. S13, one can visualize directly the differences in K_L^h spatially in such COMPEITS images.

Although COMPEITS images could be generated for 5-nm Au nanoparticles in the same way as for nanoplates and nanorods, they are less informative. The main reason is that the physical size of the particles is smaller than the ~10 nm resolution here of the single-molecule super-resolution imaging. Using 10×10 nm^2 or bigger bin sizes in the COMPEITS image gives no meaningful geometric information regarding distribution of adsorption affinity with respect to the structural contour of the particle (Supplementary Fig. 10f-h). Using bin sizes smaller than 5 nm was possible, e.g., 2×2 nm^2 bins could present the 2D distributions of the product molecules clearly (Supplementary Fig. 10b-c). However, a misleading ring pattern would show up outside the structural contour of the 5-nm nanoparticle (Supplementary Fig. 10d-e). Fitting the n-vs-[L] curves for molecules inside the structural contour or outside gives comparable K and h values, and the conclusion holds when fitting the n-vs-[L] curves for molecules inside a circle with 10 nm diameter or outside (Supplementary Fig. 10i-j). Thus, the variation of affinity suggested by the ring pattern in the COMPEITS image is not valid. This ring pattern is an artifact resulting from the mathematical manifestation of the noise level away from the contour of the nanoparticle. The n_2 values could be small (e.g., 1 or 2) for pixels away from the nanoparticle (because there is no fluorogenic catalytic reaction occurring) and thus lead to large values of $\Delta(n^{-1})$ (= $1/n_2 - 1/n_1$), i.e., over 0.5. This artifact does not affect the utility of COMPEITS images for the nanorods and nanoplates, as in those cases we compare $\Delta(n^{-1})$ for different regions within their structural contour and their sizes are much larger than the ~10-40 nm imaging resolution.

We can use the ratio of standard deviation and the mean, defined as the heterogeneity index (HI), to evaluate the spreading of data from individual particles. For CTAB adsorption on 50 of 5-nm Au nanoparticles (Fig. 1i), HI for the affinity K is 0.12/0.66 = 18%; and HI for the Hill coefficient h is 0.44/2.07 = 21%. The heterogeneity can be in part attributed to the heterogeneity of particle size: the diameter of the nominal 5-nm particles from TEM is 6.0 ± 1.6 nm,[23] where the HI is 27%.

3.2 Possible residual citrate in solution does not affect the results from 5-nm Au nanoparticles

A trace amount of citrate may exist in the solution of the commercial 5-nm Au nanoparticles we used. For the single-particle titration experiments performed in a flow cell, the citrate is expected to be washed away by the flow solution, and therefore would not affect the measurement of the target ligand. As the results from bulk titration (Supplementary Table 2a) and that from single-particle titration (Supplementary Table 2b) are comparable, the role of citrate should be minimal.

To be more stringent, we estimated the concentration of citrate in our bulk reaction mixture and tested its effect. Assuming the citrate concentration in the Au nanoparticle solution is at the upper limit of 'a trace amount', i.e., ~100 parts per million, which is roughly 0.1 g/L or ~0.1 mM. In a typical bulk titration experiment, the nanoparticle solution is diluted by ~100 fold in the reaction mixture, corresponding to a concentration in the order of 1 μM. Titration with additionally added citrate up to 10 μM, i.e., 10 times higher, only led to <10% change of the reaction rate (Supplementary Fig. 11). Consequently, it is safe to conclude that the role of citrate is negligible in the bulk titration.

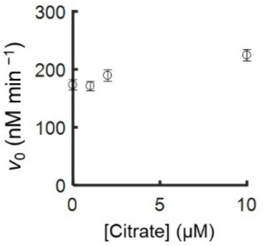

Supplementary Fig. 11 | Citrate shows negligible effect in bulk titration using 5-nm Au nanoparticles. The data were collected at $[R]_0$ = 10 µM, $[NH_2OH]_0$ = 1.0 mM, [Au nanoparticle] = 0.01 nM in 7 mM pH 7.4 phosphate buffer.

3.3 Decrease of reaction rates during COMPEITS titration is not due to catalyst deactivation

One might question whether the decrease of the reaction rates during COMPEITS titration experiments is due to catalyst deactivation over time instead of ligand competition. Our previous work showed that 6-nm pseudospherical Au nanoparticles (named so for their actual size, but is the same product as the 5-nm Au NPs used in this work) showed stable activity over the reduction of R for at least 3-4 h[23], and that Au nanorods were stable for at least 6 h[7]. Here, we verified that Au nanoplates were stable for at least 4 hours (Section 4.2). Therefore, we confirmed that the decrease of reaction rates during COMPEITS titration is not due to catalyst deactivation for all the morphologies of nanoparticles we studied in this work.

3.4 Ligand adsorption titration curves: adsorption affinity and (non)cooperativity of CTAB/CTAOH/CTAC, PVPs, halides, and thiol

Titration plots of single 5-nm Au nanoparticles from single-molecule fluorescence microscopy of the fluorogenic auxiliary reaction in the presence of increasing competing ligand concentrations are summarized in Supplementary Fig. 12 and Supplementary Table 2b. The obtained K_L and h values are consistent with the bulk titration data in Section 2.

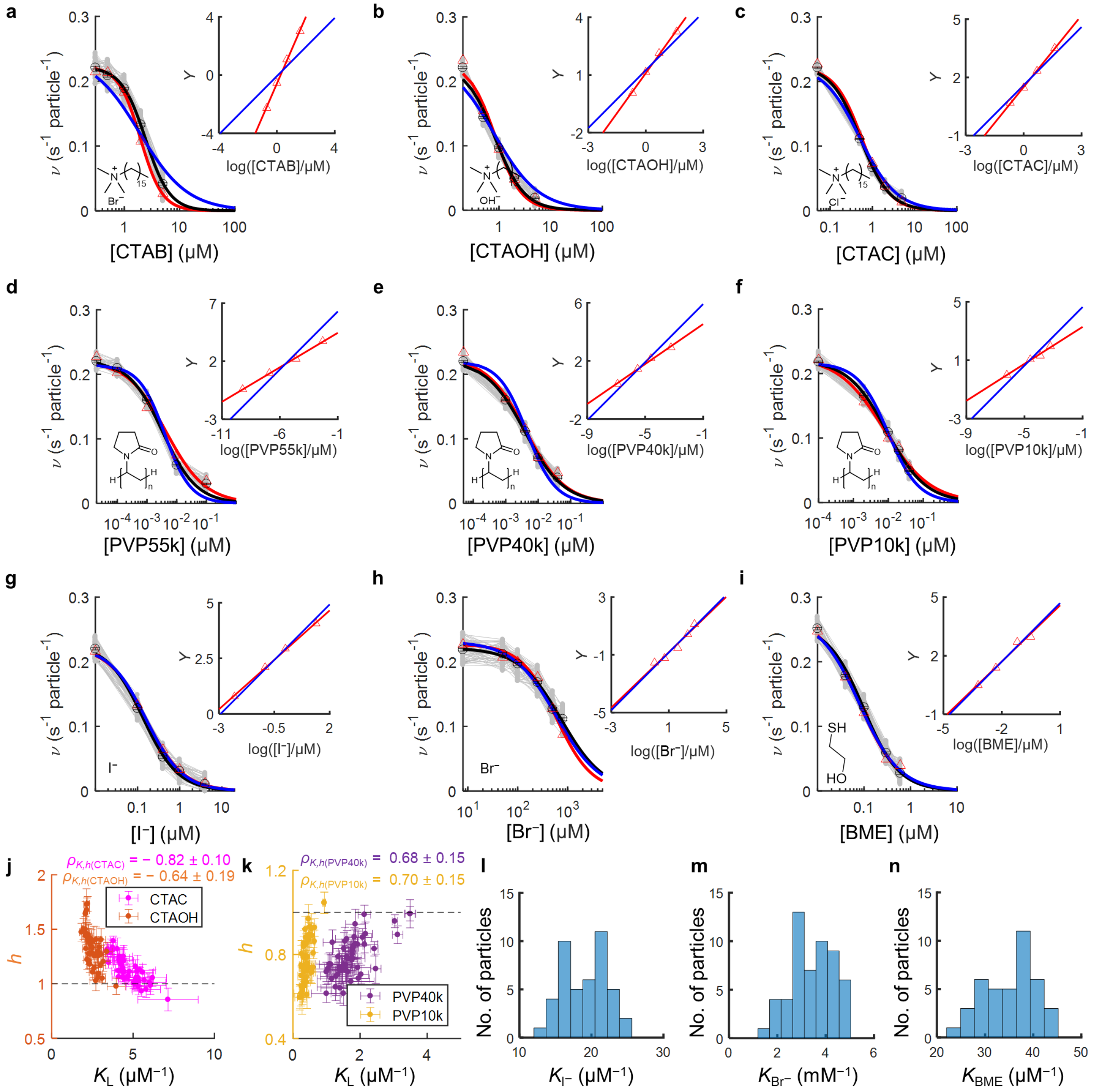

Supplementary Fig. 12 | Examples of ligand competition titration plots of single 5-nm Au nanoparticles for CTAB (a), CTAOH (b), CTAC (c), PVP55k (d), PVP40k (e), PVP10k (f), I⁻ (g), Br⁻ (h), BME (i), respectively (grey) , *h* vs. *K* plots for CTAC and CTAOH (j) and PVP40k and PVP10k (k), and histograms of *K* for I⁻ (l), Br⁻ (m) and BME (n) (Supplementary Information section 3.4). Data points at [L] = 0 are placed on the *y*-axes manually. Red triangles: a single particle example under each condition; black circles: averages among many individual particles; red/black lines: corresponding fits with Eq. (1). Blue line: Fits with h set to 1. Insets: the corresponding Hill plots of the selected single particles (points); lines: fits with the rearranged linear Hill form of Eq. (1) (Eq. S14) with h floating (red) or set to 1 (blue); the slope here is h. Error bars are s.e.m. All fitting parameters summarized in Supplementary Table 2b. **a** is the same as Fig. 1b; **d** same as Fig. 1c; and **g** same as Fig. 1d.

Comparing CTAB with CTAOH and CTAC, their adsorption affinities on 5-nm Au nanoparticles follow $K_{\mathrm{CTAB}} < K_{\mathrm{CTAOH}} < K_{\mathrm{CTAC}}$ (Fig. 1f); this order is consistent with that Br^- co-adsorbs with CTA^+ on Au surfaces, which likely weakens the columbic attraction between CTA^+ and the negatively-charged Au nanoparticle surface, whereas Cl^- barely co-adsorbs[31]. Moreover, they both show positive adsorption cooperativity ($h > 1$) like CTAB, where stronger affinity accompanies weaker cooperativity (Fig. 1f). The smaller h of CTAOH and CTAC indicates the counter anion plays a role in cooperativity, besides in affinity.

For PVP, upon decreasing the average molecular weight from 55k to 40k and 10k g/mol, the average adsorption affinity (K) deceases (Fig. 1g) and becomes unmeasurable for the monomer *N*-vinylpyrrolidone (Supplementary Fig. 9j); this strong dependence suggests that PVP adsorption on Au particle surfaces is enhanced by multivalency effects. Interestingly, their Hill coefficients h are about the same (~0.7, Fig. 1g), suggesting that the inter-chain interactions of PVP are dominated by sub-chain structural features, like thermal blobs, which are similar in size regardless of the molecular weight[32].

For halides, on average, $K_{\mathrm{I}^-} = 5.8 \pm 0.1$ μM^{-1}, larger than $K_{\mathrm{Br}^-} = 0.0034 \pm 0.0001$ μM^{-1} (Fig. 1h; the contribution of their potassium counter-cations is negligible; Section 2), corroborating a known trend[33-35].

For β-mercaptoethanol, its $K_{\mathrm{BME}} = 35 \pm 1$ μM^{-1}, comparable to those from bulk calorimetry measurements [36,37] and in which the thiol group dominates as ethanol adsorption is minimal (Supplementary Fig. 9k).

4 Supplementary results of COMPEITS imaging of ligand adsorption on single Au nanoplates

4.1 COMPEITS images indicate spatially (in)homogeneous adsorption on single nanoplates of different ligands

Each pixel of the COMPEITS image was calculated as $\Delta(n^{-1}) = 1/n_2 - 1/n_1$, where the subscript 1 and 2 represents the images with zero and high [L], respectively. As $\Delta(n^{-1}) \propto \Delta(v^{-1}) \propto K_{\mathrm{L}}^{h}$ based on Eq. (1) and Eq. S13, one can visualize directly the differences in K_{L}^{h} spatially in such COMPEITS images.

As described in the main text, we studied Au nanoplates for adsorption by six ligands, spanning positive (i.e., CTAB), negative (i.e., PVP55k and PVP10k), and no cooperativity (i.e., BME, I^-, and Br^-). Representative COMPEITS images give direct visual presentations of their preferential (or non-preferential) adsorption on different locations on single nanoplates (Supplementary Fig. 13). CTAB (Supplementary Fig. 13a), PVP55k (Supplementary Fig. 13b), and PVP10k (Supplementary Fig. 13c) adsorb more strongly at the corner and edge regions than at the flat facet region, while I^- (Supplementary Fig. 13d) and Br^- (Supplementary Fig. 13e) prefer to adsorb on the flat facet. On the other hand, BME shows no apparent preference among the regions (Supplementary Fig. 13f). Within the flat facet, all ligands except BME show a larger adsorption affinity at the center of the flat facet than at the periphery.

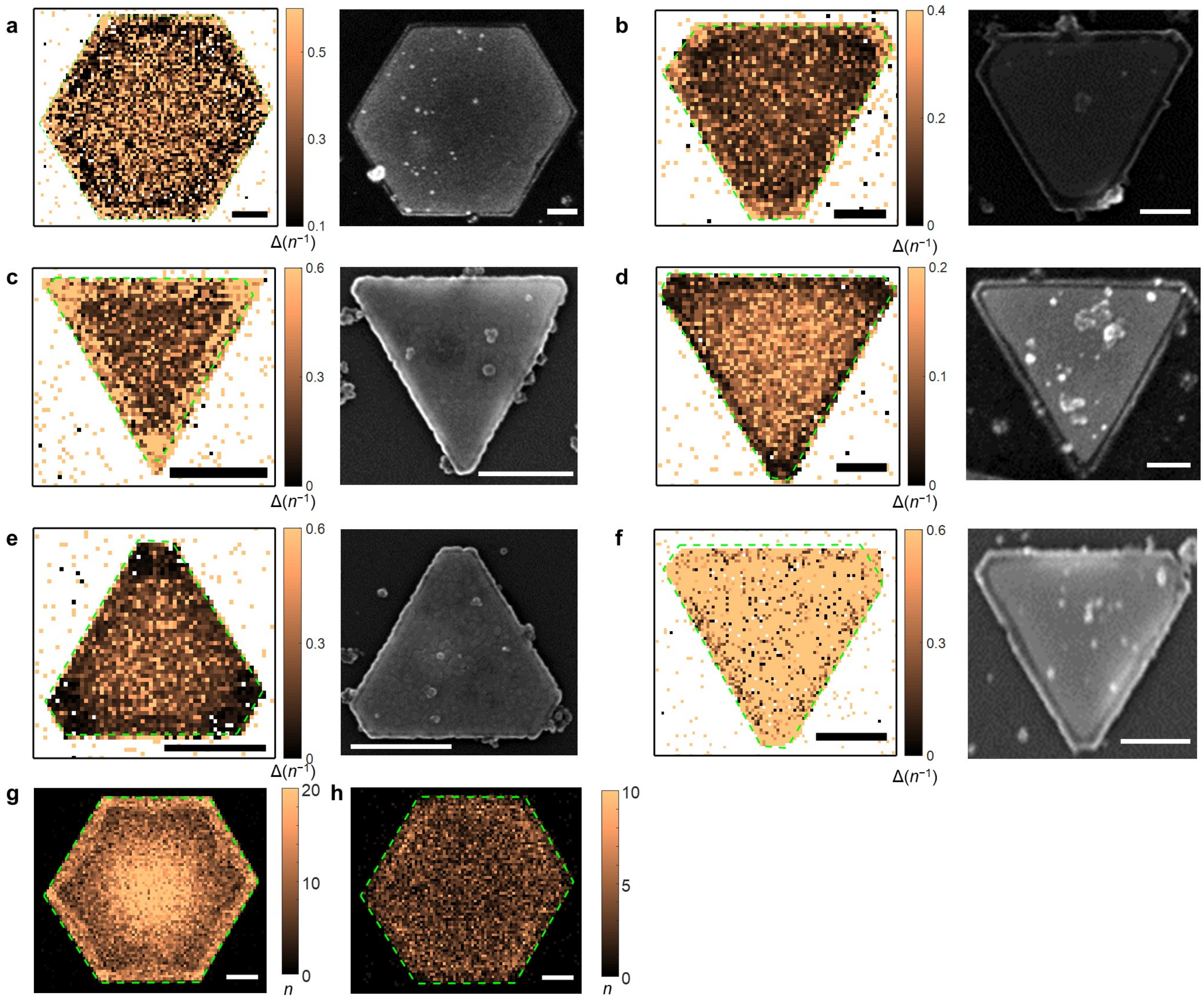

Supplementary Fig. 13 | Representative COMPEITS images showing the adsorption of different ligands on single Au nanoplates and the corresponding SEM images (Supplementary Information section 4.1)**.** The ligands and experimental conditions are: CTAB at 0 and 0.50 µM (**a**), PVP55k at 0 and 4.0 nM (**b**), PVP10k at 0 and 10 nM (**c**), I^- at 0 and 0.10 µM (**d**), Br^- at 0 and 2.0 mM (**e**), and BME at 0 and 50 nM (**f**), respectively. COMPEITS images (left panels): white/null pixels represent occasional negative values or infinities due to 1/0 calculations; pixel size: 40 × 40 nm^2 for **a**, **b**, and **d**, and otherwise 20 × 20 nm^2. The small bright objects in the SEM images (right panels) are small mesoporous silica particles adsorbed onto the mesoporous silica shell; they have no observable effect on the COMPEITS images nor the titration curves. All scale bars are 500 nm. **g**-**h**, Super resolution images of product molecules on the nanoplate shown in **a** at [CTAB] = 0 (**g**) and [CTAB] = 0.50 µM (**h**); pixel size: 40 × 40 nm^2. **a** is also presented in Fig. 2a and 2d in the main text.

4.2 The decrease in reaction rates during COMPEITS titration is not due to catalyst deactivation

A set of control experiments was performed to test the stability of the mesoporous silica coated Au nanoplates under the imaging conditions. As described earlier, the nanoplates were first titrated at increasing concentrations of R, and then at increasing concentrations of the ligand at the highest concentration of R. The red curve in Supplementary Fig. 14 shows the change of averaged reaction rate of all nanoplates $\langle v_{NP} \rangle$ within 0.5 h in the first flow cell as the titration progressed. The black curve shows the progress of $\langle v_{NP} \rangle$ from nanoplates in the second flow cell (prepared with the same batched of nanoplates), where the data points starting at time = 2 h were all collected at [R] = 0.1 µM with no CTAB. Both flow cells went through the same titration conditions before time = 2 h, and the $\langle v_{NP} \rangle$ at each condition were the same within errors before time = 2 h (not shown in the figure for brevity). The black curve clearly shows

that without the introduction of CTAB, the nanoplates remain stable for at least 4 hours, within the duration of a set of COMPEITS titration experiments.

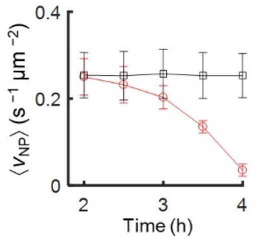

Supplementary Fig. 14 | Control experiments for the stability of Au nanoplates over time. Data on the red curve was a part of a typical titration experiment, where [R] was held at 0.1 μM, [NH_2OH] was 1 mM, and [CTAB] is 0, 0.5, 1, 2, and 5 μM, respectively. Points on the black curve were all collected at the same solution with 0.1 μM of R and 1 mM of NH_2OH. Each point represents the average activity of ~ 30 nanoplates within 0.5 h. Error bars are s.d.

4.3 Additional statistical plots of *K* and *h* of ligand adsorption on single nanoplates reveal sub-particle and sub-facet differences

As described in Section 1.5, fitting the titration curve of a region of a single nanoplate gives the corresponding K and h values of the corresponding region. In this way, the K vs. h correlation plot from multiple nanoplates could be obtained for a ligand showing cooperativity (Supplementary Fig. 15). The correlations of K and h, as well as the distributions of K and h at different regions can be seen for CTAB, PVP55k, and PVP10k (Supplementary Fig. 15a-c). As for I^-, Br^-, and BME, h is fixed to 1, so only distributions of K are shown (Supplementary Fig. 15d-f). The mean and the standard error of the mean for these values, as well as the Pearson's cross correlation coefficients, are summarized in Supplementary Table 3. Pearson's cross-correlation coefficient $\rho(x,y)$ is a measure of the strength and direction of the linear relationship between two variables x and y. It can be calculated by the following equation:

$$\rho(x,y) = \frac{\sum_{i=1}^{n}(x_i - \langle x \rangle)((y_i - \langle y \rangle))}{\sqrt{\sum_{i=1}^{n}(x_i - \langle x \rangle)^2}\sqrt{\sum_{i=1}^{n}(y_i - \langle y \rangle)^2}} \quad \text{Eq. S20}$$

where n is the sample size, $\langle \ \rangle$ denotes averaging. Thus, ρ is essentially a normalized measurement of the covariance, and always has a value between −1 and 1: $\rho(x,y) = 1$ implies that x and y can be perfectly described by a linear equation, with all data points lying on a line for which y increases as x increases; $\rho(x,y) = -1$ implies that all data points lie on a line for which y decreases as x increases; $\rho(x,y) = 0$ implies that there is no linear correlation between the variables.

Supplementary Table 3 | Summary of average values and their cross correlation coefficients of *K* and *h* of different ligands at different regions of nanoplates. Parts of these data are plotted in Fig. 2. Errors of K and h are s.e.m.; errors of cross correlation coefficients are 95% confidence bounds.

Ligand	No. of nanoplates measured	K_c (M^{-1})	K_e (M^{-1})	K_f (M^{-1})	h_c	h_e	h_f	$\rho(K_c, h_c)$	$\rho(K_e, h_e)$	$\rho(K_f, h_f)$
PVP55k	40	1.3 ± 0.2 (×10⁹)	6.2 ± 1.4 (×10⁸)	3.7 ± 1.3 (×10⁸)	0.84 ± 0.10	0.77 ± 0.08	0.68 ± 0.09	0.77 ± 0.16	0.85 ± 0.04	0.86 ± 0.10
PVP10k	30	4.7 ± 0.4 (×10⁸)	4.2 ± 0.5 (×10⁸)	3.2 ± 0.4 (×10⁸)	0.90 ± 0.05	0.80 ± 0.05	0.76 ± 0.03	0.87 ± 0.10	0.86 ± 0.03	0.89 ± 0.08
BME	40	4.9 ± 0.4 (×10⁷)	4.8 ± 0.4 (×10⁷)	4.8 ± 0.4 (×10⁷)	1	1	1	N/A	N/A	N/A
I^-	36	5.2 ± 0.3 (×10⁶)	5.8 ± 0.5 (×10⁶)	6.4 ±0.5 (×10⁶)	1	1	1	N/A	N/A	N/A
CTAB	55	9.1 ± 0.3 (×10⁵)	7.8 ± 0.3 (×10⁵)	5.4 ± 0.3 (×10⁵)	1.7 ± 0.1	1.8 ± 0.1	2.2 ± 0.1	−0.36 ± 0.25	−0.47 ± 0.21	−0.42 ± 0.23
Br^-	35	1.3 ± 0.1 (×10³)	1.6 ± 0.1 (×10³)	2.8 ± 0.2 (×10³)	1	1	1	N/A	N/A	N/A
(Cont.)										
**Ligand**	**No. of nanoplates measured**	K_i (M^{-1})	K_m (M^{-1})	K_o (M^{-1})	h_i	h_m	h_o	$\rho(K_i, h_i)$	$\rho(K_m, h_m)$	$\rho(K_o, h_o)$

PVP55k	40	4.8 ± 0.9 (×10^8)	3.7 ± 1.0 (×10^8)	2.6 ± 0.7 (×10^8)	0.59 ± 0.09	0.72 ± 0.08	0.76 ± 0.09	0.73 ± 0.16	0.62 ± 0.21	0.58 ± 0.23
PVP10k	30	3.5 ± 0.3 (×10^8)	3.2 ± 0.3 (×10^8)	2.8 ± 0.3 (×10^8)	0.72 ± 0.05	0.79 ± 0.05	0.87 ± 0.04	0.78 ± 0.14	0.86 ± 0.10	0.85 ± 0.10
BME	40	4.9 ± 0.5 (×10^7)	4.6 ± 0.4 (×10^7)	4.4 ± 0.4 (×10^7)	1	1	1	N/A	N/A	N/A
I^-	36	6.9 ± 0.4 (×10^6)	6.4 ± 0.4 (×10^6)	5.9 ± 0.4 (×10^6)	1	1	1	N/A	N/A	N/A
CTAB	55	6.8 ± 0.3 (×10^5)	5.4 ± 0.3 (×10^5)	4.0 ± 0.2 (×10^5)	2.4 ± 0.1	2.2 ± 0.1	2.0 ± 0.1	−0.45 ± 0.22	−0.46 ± 0.22	−0.38 ± 0.24
Br^-	35	3.1 ± 0.2 (×10^3)	2.8 ± 0.2 (×10^3)	2.1 ± 0.2 (×10^3)	1	1	1	N/A	N/A	N/A

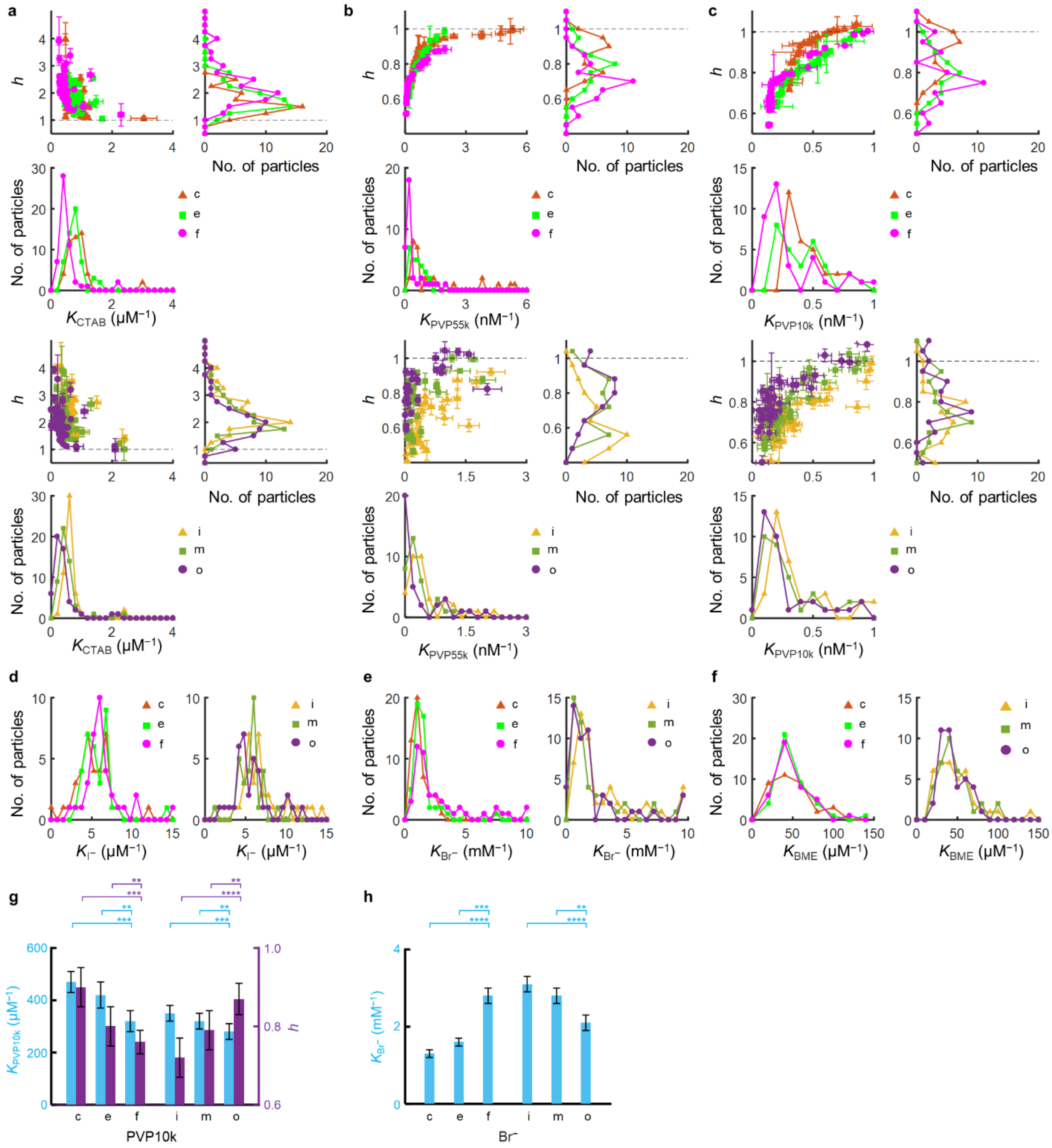

Supplementary Fig. 15 | Distributions of *K* (and *h*) of different ligands at different regions of Au nanoplates (Supplementary Information section 4.3)**.** The scatter plots and histograms are included for ligands showing cooperativity: CTAB (**a**), PVP55k (**b**),

and PVP10k (**c**); only the histograms of *K* are shown for I^- (**d**), Br^- (**e**), and BME (**f**) as they do not show cooperativity (i.e., $h = 1$). The mean and s.e.m. are listed in Supplementary Table 3. **a** (first row, left) and **b** (first row, left) are presented in Fig. 2h and i, respectively. **g**-**h**, Facet and sub-facet differences in adsorption affinity (*K*) and cooperativity (*h*) of PVP10k (**g**) and Br^- (**h**, no cooperativity) on 30 and 35 nanoplates, respectively. $^{**}p < 0.01$; $^{***}p < 0.001$; $^{****}p < 0.0001$; paired Student's *t* test. Error bars in **a**-**c** are s.d. from titration curve fitting, s.e.m. in **g**-**h**.

4.4 Particle-averaged titration analyses also identify sub-particle differences in ligand adsorption

In addition to the single-particle analysis, an alternative way to analyze the data from multiple nanoplates is to obtain $\langle v \rangle$ ($\langle\ \rangle$ denotes averaging) from all the nanoplates studied and then fit the $\langle v \rangle$ - [L] plot (Supplementary Fig. 16 and Supplementary Table 4). The trends of *K* (and *h* for CTAB and PVP) can also be directly seen for corner/edge/flat-facet regions (Supplementary Fig. 16A-C), confirming the variations of adsorption behaviors at different facets on nanoplates. The values of *K* and *h* obtained from fitting the particle-averaged titration curve (Supplementary Table 4) are comparable to those from single-particle analysis (Supplementary Table 3).

However, the particle-averaged analysis of in/mid/out sub-facet regions do not produce clear differences in *K* and *h* (Supplementary Fig. 16Af-Aj). One reason is that different sized particles have different gradients (i.e., heterogeneity among individual particles). Therefore, the trends of *K* and *h* for the inner/middle/outer sub-facet regions are washed out and masked in the particle-averaged analysis. This result highlights the advantages of single-particle imaging, which allows for single-particle analysis.

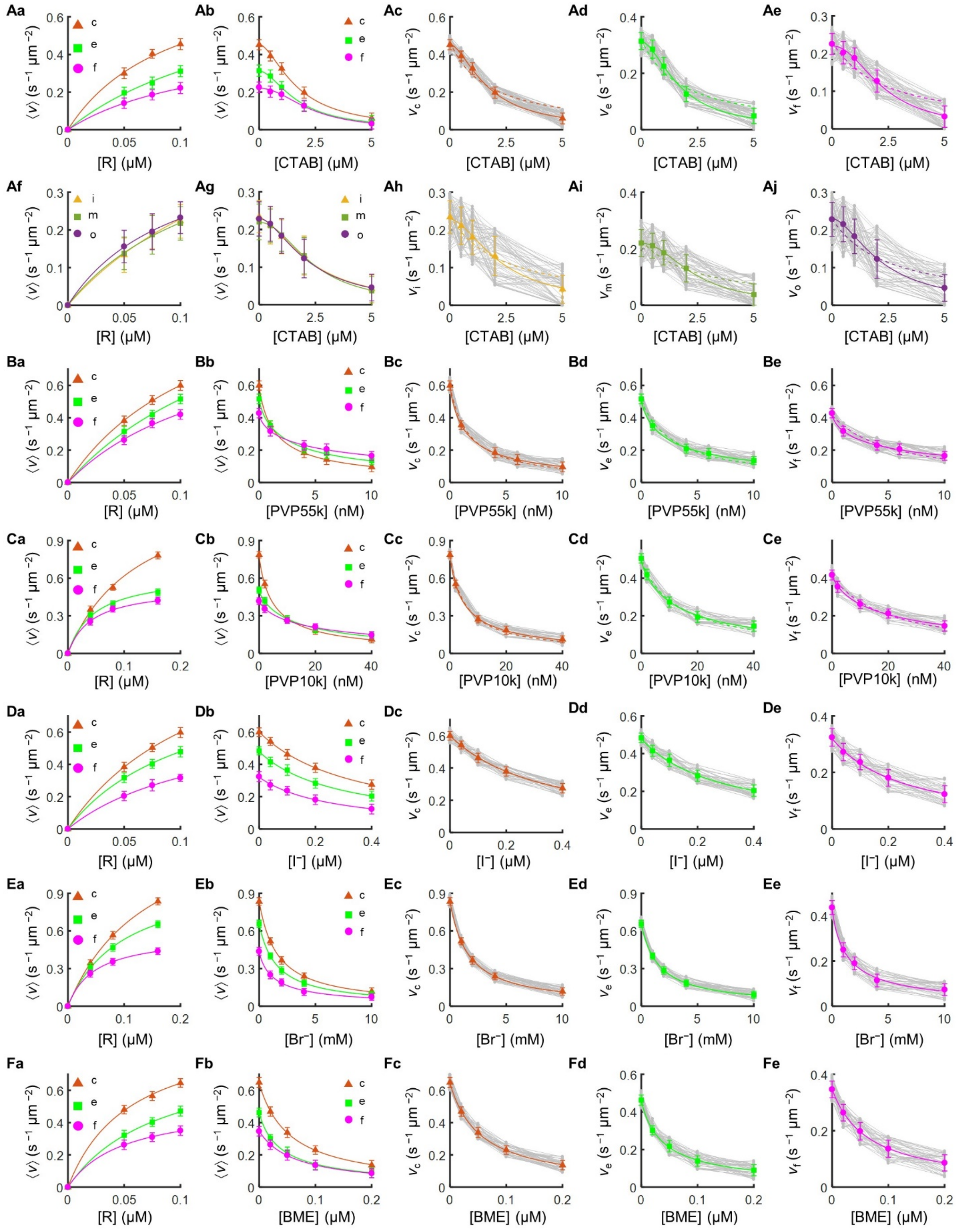

Supplementary Fig. 16 | Particle-averaged, spatially resolved, titration analyses for ligand adsorption on nanoplates. (**Aa-Ab**) The particle-averaged $\langle v \rangle$ - [R] curve (Aa) and the corresponding $\langle v \rangle$ - [CTAB] curve (Ab) of the corner, edge, and flat facet regions. (Ac) The v_c - [CTAB] titration plots for the corner regions of all single nanoplates (grey). Colored triangles, solid line, and

dash line: particle-averaged data, the fit with Eq. S11, and the fit with Eq. S11 with h fixed to 1, respectively. (**Ad**-**Ae**) Similar to Ac, but for the edge region (Ad) and for the flat facet region (Ae). (Af-Aj) Similar to Aa-Ae, but for the inner, middle, and outer regions. (**B**-**F**) Similar to Aa-Ae, but for PVP55k (B), PVP10k (C), I^- (D), Br^- (E), and BME (F), respectively. As such particle-averaged analysis cannot effectively differentiate the K and h for inner/middle/outer sub-facet regions as shown in Af-Aj for CTAB, similar plots for other ligands are omitted. The corresponding fitting parameters are listed in Supplementary Table 4. All error bars are s.d.

Supplementary Table 4 | List of fitting parameters of particle-averaged titration curves of adsorption of different ligands on nanoplates shown in Supplementary Fig. 16. Errors are s.d. from fitting.

**Ligand**	K_c (M^{-1})	K_e (M^{-1})	K_f (M^{-1})	h_c	h_e	h_f
PVP55k	1.4 ± 0.1 (×10^9)	6.4 ± 0.2 (×10^8)	4.0 ± 0.4 (×10^8)	0.83 ± 0.02	0.75 ± 0.01	0.68 ± 0.05
PVP10k	5.1 ± 0.3 (×10^8)	4.6 ± 0.4 (×10^8)	3.1 ± 0.6 (×10^8)	0.89 ± 0.02	0.84 ± 0.03	0.76 ± 0.05
BME	5.1 ± 0.4 (×10^7)	4.8 ± 0.3 (×10^7)	4.8 ± 0.2 (×10^7)	1	1	1
I^-	5.2 ± 0.3 (×10^6)	5.8 ± 0.5 (×10^6)	6.4 ±0.5 (×10^6)	1	1	1
CTAB	9.3 ± 0.8 (×10^5)	7.6 ± 0.6 (×10^5)	5.8 ± 0.3 (×10^5)	1.7 ± 0.2	1.8 ± 0.2	2.2 ± 0.2
Br^-	1.3 ± 0.1 (×10^3)	1.6 ± 0.1 (×10^3)	2.8 ± 0.2 (×10^3)	1	1	1
(Cont.)						
**Ligand**	K_i (M^{-1})	K_m (M^{-1})	K_o (M^{-1})	h_i	h_m	h_o
CTAB	5.6 ± 1.3 (×10^5)	5.5 ± 1.5 (×10^5)	6.9 ± 1.8 (×10^5)	2.1 ± 0.7	2.0 ± 0.8	1.8 ± 0.6

4.5 Adsorption strength and cooperativity vs. nanoplate size and shape

With the data available, we looked into the potential impacts of the sizes or shapes of the nanoplates on the ligand adsorption behaviors. To that end, the K or h of individual particles/regions is plotted against the radius (i.e., the average distance from the center to the vertex) or the shape factor (i.e., the ratio of the length sum of the shorter three edges to the length sum of the longer three from the view of a hexagon, which is 0 for a triangle and 1 for a regular hexagon (see Supplementary Fig. 1e for distributions), and the corresponding Pearson's cross correlation coefficients are calculated (e.g., Supplementary Fig. 17 and Supplementary Table 5 on CTAB, PVP55k, and I^-).

Within the errors of the Pearson's cross correlation coefficients, K or h of the corner and edge regions show no clear correlation with the size or shape of the nanoplates (e.g., Supplementary Fig. 17Aa-Ad). We attribute this to that the size of the nanoplates exceeds the range where the size plays a significant role.

On the other hand, adsorption affinities on the overall flat facet region or the inner, middle, and outer sub-facet regions correlate negatively with the particle sizes (e.g., Supplementary Fig. 17Ae, Ag). We previously established that on the flat facet, the structural defects decrease in density from the center toward the periphery because of their seeded growth mechanism[2]. Therefore, the size effect in this case can be attributed to the differences in density of structural defects. The shape of the nanoplates has no observable effect on the ligand adsorption behaviors on the inner, middle, and outer sub-facet regions (e.g., Supplementary Fig. 17Af, Ah).

The corresponding plots for PVP10k, Br^-, and BME look similar to those shown in Supplementary Fig. 17 and thus omitted to avoid redundancy.

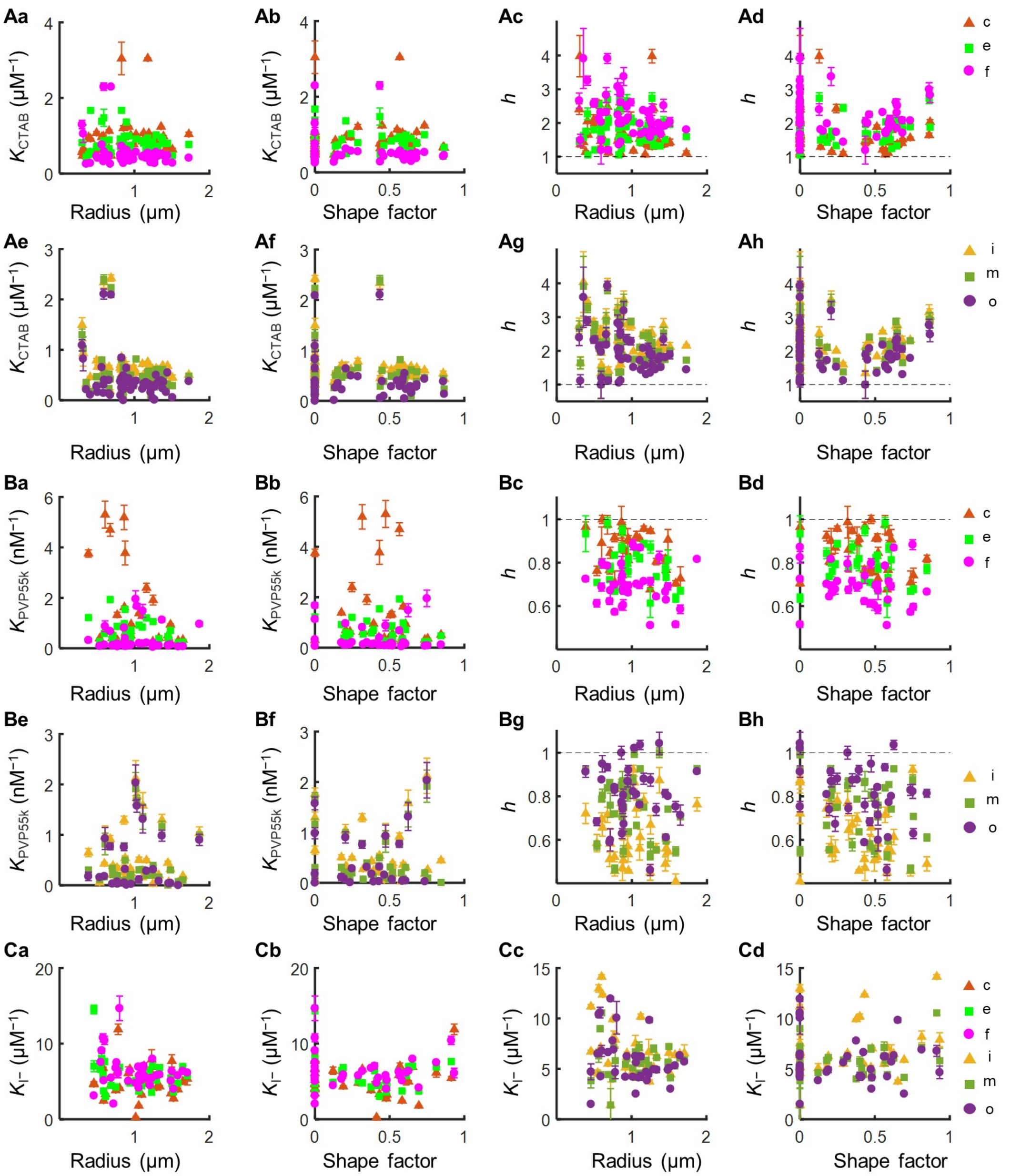

Supplementary Fig. 17 | Effects of the size and shape of nanoplates on the K and h of ligand adsorption. (**Aa**-**Ad**) Correlation plots of K_{CTAB} - radius (Aa), K_{CTAB} - shape factor (Ab), radius - h (Ac), and shape factor - h (Ad) for CTAB adsorption at the corner, edge, and flat facet regions of individual nanoplates. (**Ae**-**Ah**) Similar to Aa-Ad, but for CTAB adsorption at the inner, middle, and outer regions. (**B**-**C**) Similar to A, but for the adsorption of PVP55k (B) and I^- (C).

Supplementary Table 5 | List of Pearson's cross correlation coefficients for K and h vs. the radius or the shape factor. Errors are 95% confidence bounds.

**CTAB**	K_c	K_e	K_f	h_c	h_e	h_f
**radius**	0.17 ± 0.27	−0.16 ± 0.26	−0.31 ± 0.25	−0.20 ± 0.27	−0.06 ± 0.25	−0.33 ± 0.25
**shape factor**	0.08 ± 0.28	−0.05 ± 0.27	−0.09 ± 0.27	−0.25 ± 0.26	−0.01 ± 0.27	−0.12 ± 0.27
**CTAB**	K_i	K_m	K_o	h_i	h_m	h_o
**radius**	−0.28 ± 0.25	−0.26 ± 0.26	−0.29 ± 0.25	−0.30 ± 0.25	−0.34 ± 0.24	−0.33 ± 0.25
**shape factor**	−0.08 ± 0.27	−0.11 ± 0.27	−0.09 ± 0.27	−0.13 ± 0.27	−0.12 ± 0.27	−0.12 ± 0.27

(Cont.)

**PVP55k**	K_c	K_e	K_f	h_c	h_e	h_f
**radius**	−0.01 ± 0.33	0.06 ± 0.33	0.04 ± 0.33	−0.10 ± 0.33	−0.15 ± 0.33	−0.04 ± 0.33

**shape factor**	−0.10 ± 0.33	−0.11 ± 0.33	−0.08 ± 0.33	−0.36 ± 0.30	−0.31 ± 0.30	−0.34 ± 0.30
**PVP55k**	$\boldsymbol{K_i}$	$\boldsymbol{K_m}$	$\boldsymbol{K_o}$	$\boldsymbol{h_i}$	$\boldsymbol{h_m}$	$\boldsymbol{h_o}$
**radius**	−0.38 ± 0.31	−0.28 ± 0.35	0.06 ± 0.33	−0.36 ± 0.32	−0.35 ± 0.33	−0.07 ± 0.33
**shape factor**	−0.15 ± 0.35	−0.15 ± 0.37	−0.08 ± 0.33	−0.25 ± 0.34	−0.20 ± 0.36	−0.16 ± 0.33

(Cont.)

$\boldsymbol{I^-}$	$\boldsymbol{K_c}$	$\boldsymbol{K_e}$	$\boldsymbol{K_f}$	$\boldsymbol{K_i}$	$\boldsymbol{K_m}$	$\boldsymbol{K_o}$
**radius**	−0.07 ± 0.33	−0.30 ± 0.32	−0.25 ± 0.32	−0.42 ± 0.28	0.00 ± 0.35	−0.27 ± 0.21
**shape factor**	0.01 ± 0.33	−0.15 ± 0.34	0.00 ± 0.34	0.18 ± 0.33	0.22 ± 0.34	−0.13 ± 0.33

5 Supplementary results of COMPEITS imaging of ligand adsorption on single Au nanorods

5.1 Additional statistical plots of *K* and *h* of ligand adsorption on single nanorods reveal sub-particle and sub-facet differences

In parallel to the analysis of ligand adsorption on nanoplates discussed in Section 4.3, *K* and *h* values of different regions of single nanorods can be obtained from fitting the titration curve of the corresponding region. The resultant *K* vs. *h* correlation plots and distributions are shown for CTAB and PVP55k (Supplementary Fig. 18a-b), and the distributions of *K* are shown for I^- and Br^- (Supplementary Fig. 18c-d). The mean and standard error of the mean for these values and the Pearson's cross correlation coefficients are summarized in Supplementary Table 6.

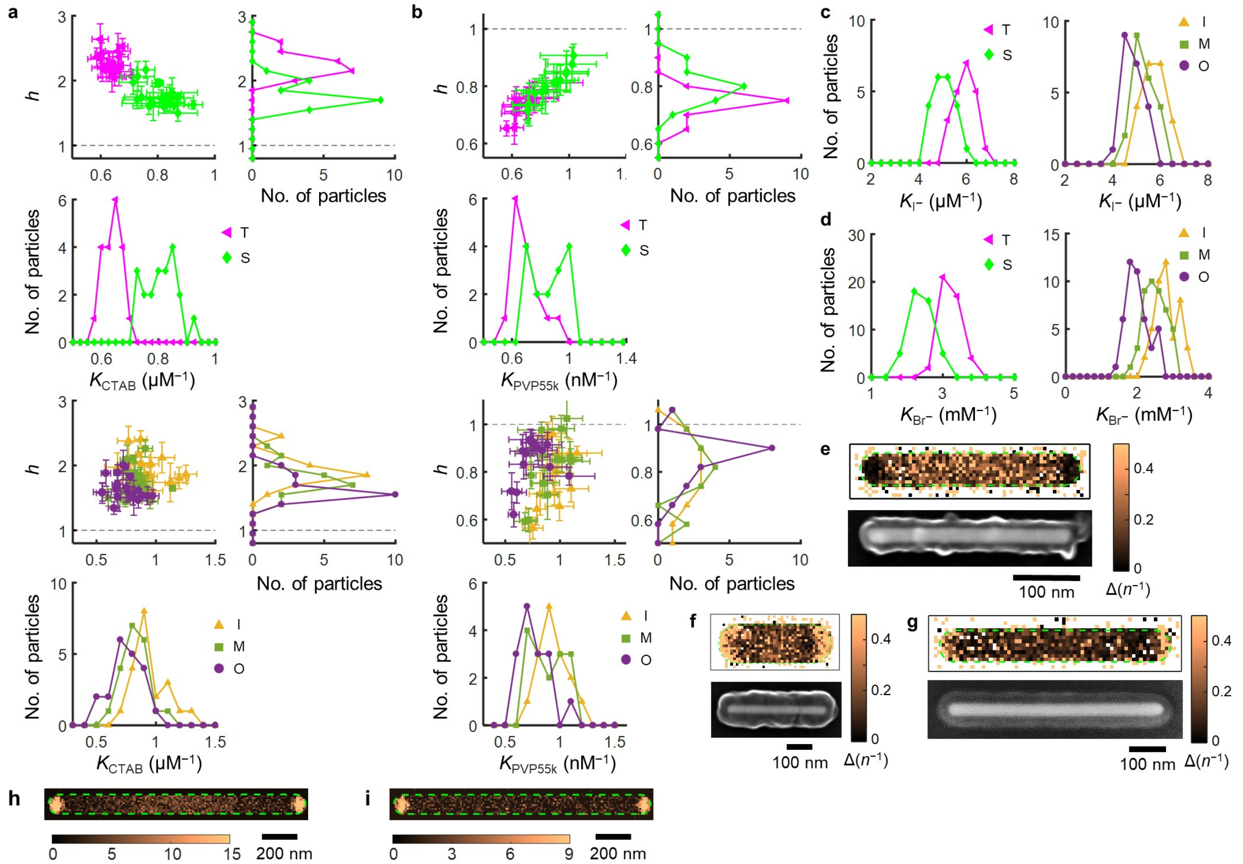

Supplementary Fig. 18 | Distributions of *K* (and *h*) of different ligands at different regions of nanorods and additional COMPEITS images. The correlation plots and histograms are included for ligands showing cooperativity: CTAB (**a**) and PVP55k (**b**); only the histograms of *K* are shown for I^- (**c**), and Br^- (**d**), which do not show cooperativity (i.e., $h = 1$). The mean and s.e.m. are listed in Supplementary Table 6. Error bars are s.d. in **a-b** from fitting. **e-g**, Representative COMPEITS images (top) showing the adsorption of different ligands on nanorods and the corresponding SEM images (bottom). The ligands and experimental conditions are PVP55k at 0 and 10 nM (**e**), I^- at 0 and 0.10 μM (**f**), and Br^- at 0 and 0.20 mM (**g**), respectively. COMPEITS images: white/null pixels represent occasional negative values or infinities due to 1/0 calculations values; pixel size: 10 × 10 nm^2. **h-i**, Super

resolution images of product molecules on the nanorod shown in Fig. 3a at [CTAB] = 0 (**h**) and [CTAB] = 0.50 μM (**i**); pixel size: 10 × 10 nm^2. Parts of **a** and **b** are also shown in Fig. 3.

Supplementary Table 6 | Summary of statistics of *K* and *h* of different ligands at different regions of nanorods. Errors of *K* and *h* are s.e.m.; errors of cross correlation coefficients are 95% confidence bounds.

Ligand	No. of nanorods	K_T (M^{-1})	K_S (M^{-1})	h_T	h_S	$\rho(K_T, h_T)$	$\rho(K_S, h_S)$
PVP55k	15	6.9 ± 0.9 (×10^8)	8.6 ± 1.2 (×10^8)	0.73 ± 0.04	0.80 ± 0.04	0.80 ± 0.12	0.88 ± 0.03
I^-	21	6.3 ± 0.5 (×10^6)	5.3 ± 0.5 (×10^6)	1	1	N/A	N/A
CTAB	20	6.4 ± 0.3 (×10^5)	8.1 ± 0.6 (×10^5)	2.3 ± 0.2	1.8 ± 0.2	−0.30 ± 0.21	−0.51 ± 0.17
Br^-	44	3.2 ± 0.1 (×10^3)	2.4 ± 0.1 (×10^3)	1	1	N/A	N/A

(Cont.)

Ligand	No. of nanorods	K_I (M^{-1})	K_M (M^{-1})	K_O (M^{-1})	h_I	h_M	h_O	$\rho(K_I, h_I)$	$\rho(K_M, h_M)$	$\rho(K_O, h_O)$
PVP55k	15	9.3 ± 0.6 (×10^8)	8.9 ± 0.7 (×10^8)	7.6 ± 0.7 (×10^8)	0.76 ± 0.07	0.82 ± 0.06	0.86 ± 0.06	0.58 ± 0.19	0.59 ± 0.18	0.46 ± 0.21
I^-	21	5.8 ± 0.3 (×10^6)	5.5 ± 0.3 (×10^6)	5.0 ± 0.3 (×10^6)	1	1	1	N/A	N/A	N/A
CTAB	20	8.7 ± 0.4 (×10^5)	8.3 ± 0.3 (×10^5)	7.6 ± 0.2 (×10^5)	1.9 ± 0.1	1.7 ± 0.1	1.6 ± 0.1	−0.40 ± 0.19	0.02 ± 0.22	−0.24 ± 0.21
Br^-	44	2.8 ± 0.2 (×10^3)	2.5 ± 0.2 (×10^3)	2.0 ± 0.2 (×10^3)	1	1	1	N/A	N/A	N/A

5.2 Particle-averaged titration analyses also identify sub-particle differences in ligand adsorption

Particle-averaged titration analyses are also performed for nanorods. Similar to the cases of nanoplates, the fitting results from the particle-averaged titration curves can effectively distinguish *K* (and *h*) from the different regions, i.e., the tip vs. the side facet regions, but did not discern clearly among the regions within the side facets, i.e., the IN, MID, and OUT regions (Supplementary Fig. 19 and Supplementary Table 7). The interpretation is the same as in Section 4.4.

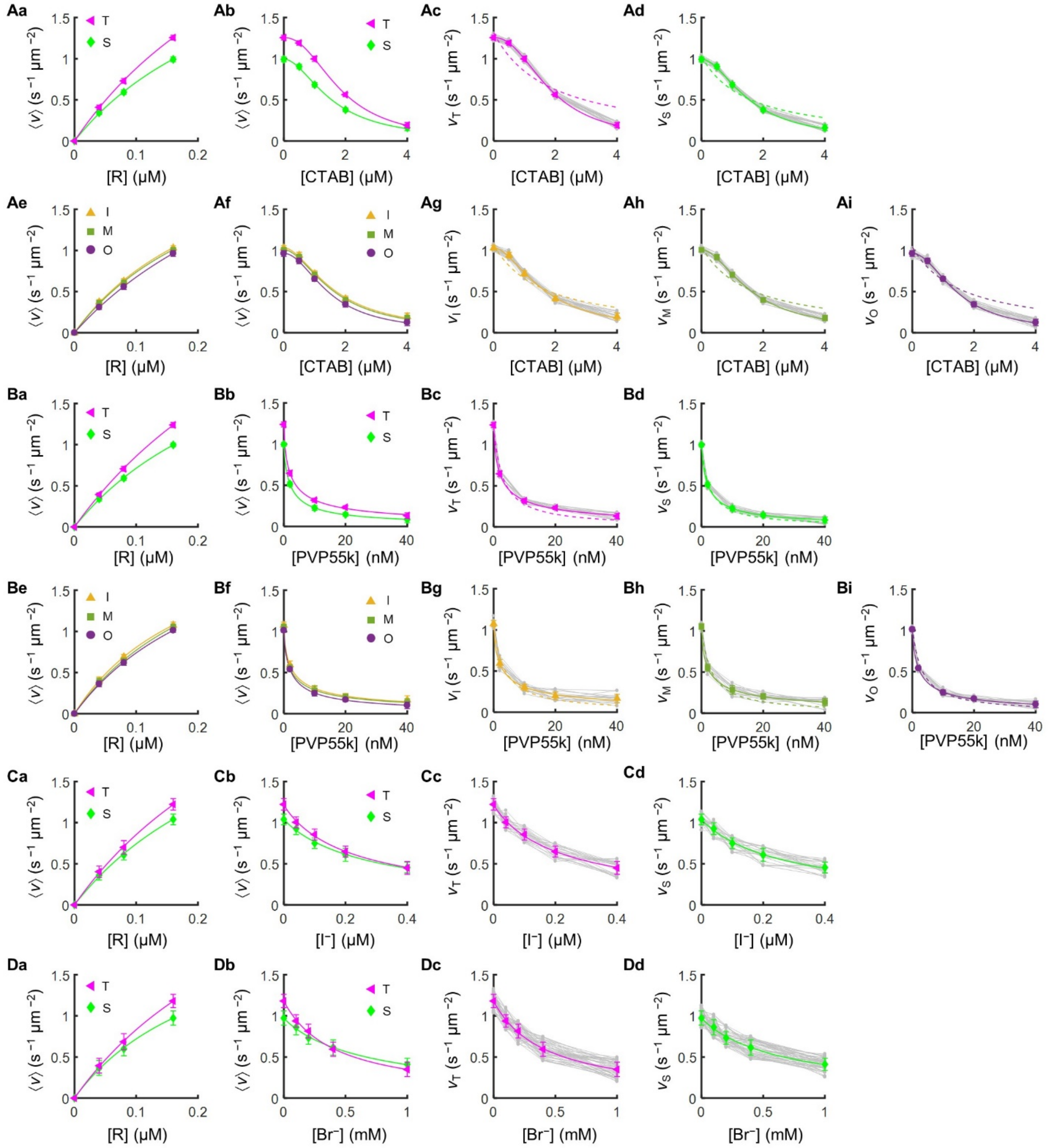

Supplementary Fig. 19 | Particle-averaged titration analyses for ligand adsorption on nanorods. (**Aa**-**Ab**) The particle-averaged ⟨v⟩ - [R] curve (Aa) and the corresponding ⟨v⟩ - [CTAB] curve (Ab) of the tip and side regions. (**Ac**) The v_T - [CTAB] titration plots for the tip regions of all single nanorods (grey). Colored triangles, solid line, and dash line: particle-averaged data, the fit with Eq. S11, and the fit with Eq. S11 with h fixed to 1, respectively. (Ad) Similar to Ac, but for the side region. (Ae-Ai) Similar to Aa-Ad, but for the IN, MID, and OUT regions. (**B**-**F**) Similar to A for PVP55k. (C-D) Similar to Aa-Ad for I^- (C) and Br^- (D), respectively. The corresponding fitting parameters are listed in Supplementary Table 7. All error bars are s.d.

Supplementary Table 7 | List of fitting parameters of particle-averaged titration curves of adsorption of different ligands on nanorods shown in Supplementary Fig. 19. Errors are s.d. from fitting.

Ligand	K_T (M^{-1})	K_S (M^{-1})	h_T	h_S		
PVP55k	6.8 ± 0.4 (×10^8)	7.6 ± 0.4 (×10^8)	0.71 ± 0.03	0.81 ± 0.05		
I^-	6.2 ± 0.5 (×10^6)	5.2 ± 0.5 (×10^6)	1	1		
CTAB	6.3 ± 0.6 (×10^5)	8.0 ± 0.3 (×10^5)	2.2 ± 0.2	1.8 ± 0.2		
Br^-	3.4 ± 0.4 (×10^3)	2.5 ± 0.2 (×10^3)	1	1		
(Cont.)						
**Ligand**	K_I (M^{-1})	K_M (M^{-1})	K_O (M^{-1})	h_I	h_M	h_O
PVP55k	8.8 ± 0.1 (×10^8)	8.9 ± 0.2 (×10^8)	7.8 ± 0.4 (×10^8)	0.68 ± 0.22	0.67 ± 0.24	0.77 ± 0.21
CTAB	8.1 ± 1.6 (×10^5)	8.1 ± 1.7 (×10^5)	8.0 ± 1.9 (×10^5)	1.9 ± 0.6	1.9 ± 0.7	2.0 ± 0.7

5.3 Adsorption strength and cooperativity vs. nanorod size

To explore the effects of size on the ligand adsorption on nanorods, we chose two parameters as the descriptors: D_T, the diameter of the semicircle of the tip region, which is also an effective measure of the width of the nanorod, and L_S, the length of the side region, where the total length of a nanorod is D_T + L_S. The correlation plots of these two parameters against K and h are shown in Supplementary Fig. 20, with the Pearson's cross correlation coefficients listed in Supplementary Table 8. For CTAB, PVP55k, and I^-, K_T correlates negatively with D_T, attributable to more under-coordinated sites available at smaller tip regions. No other clear correlations were observed.

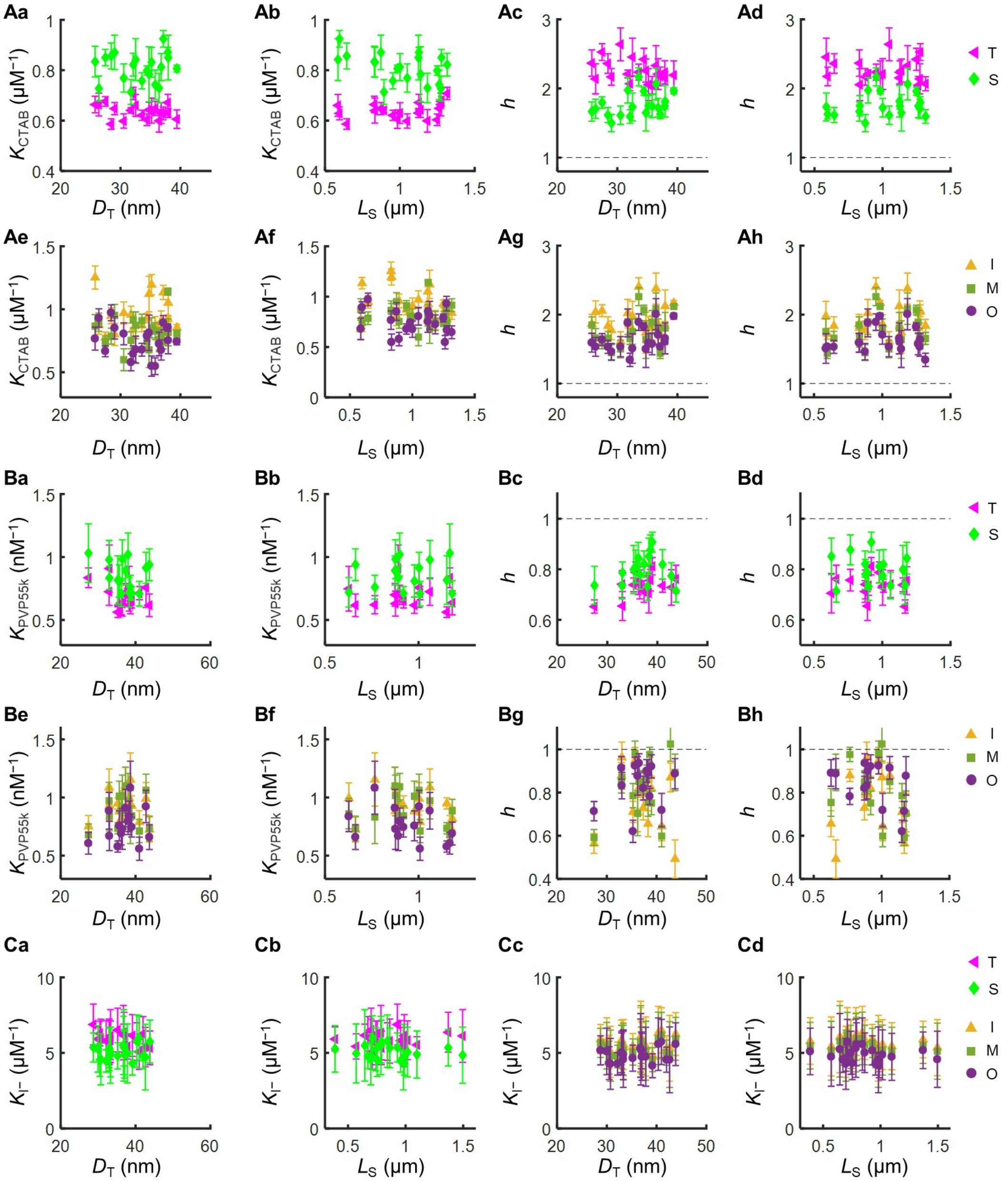

Supplementary Fig. 20 | Effects of the size and shape of nanorods on the *K* and *h* of ligand adsorption. (**Aa-Ad**) Correlation plots of K_{CTAB} - D_{T} (Aa), K_{CTAB} - L_{S} (Ab), D_{T} - h (Ac), and L_{S} - h (Ad) for CTAB adsorption at the tip and side regions. (Ae-Ah) Similar to Aa-Ad, but for CTAB adsorption at the IN, MID, and OUT regions. (**B-C**) Similar to A, but for the adsorption of PVP55k (B) and I^- (C).

Supplementary Table 8 | List of Pearson's cross correlation coefficients for *K* and *h* vs. the size parameters D_{T} and L_{S}. Errors are 95% confidence bounds.

CTAB	K_{T}	K_{S}	h_{T}	h_{S}		
D_{T}	−0.27 ± 0.21	0.08 ± 0.22	−0.37 ± 0.20	0.16 ± 0.20		
L_{S}	0.17 ± 0.21	−0.26 ± 0.19	−0.10 ± 0.22	0.19 ± 0.22		
**CTAB**	K_{I}	K_{M}	K_{O}	h_{I}	h_{M}	h_{O}
D_{T}	0.03 ± 0.23	0.04 ± 0.23	−0.24 ± 0.22	0.15 ± 0.22	0.13 ± 0.21	0.19 ± 0.20
L_{S}	−0.24 ± 0.22	0.01 ± 0.23	−0.28 ± 0.22	0.16 ± 0.22	0.19 ± 0.22	0.10 ± 0.22
(Cont.)						

PVP55k	K_T	K_S	h_T	h_S		
D_T	−0.40 ± 0.23	−0.30 ± 0.24	0.52 ± 0.20	0.18 ± 0.25		
L_S	0.03 ± 0.26	0.09 ± 0.26	−0.10 ± 0.26	−0.12 ± 0.26		
**PVP55k**	K_I	K_M	K_O	h_I	h_M	h_O
D_T	0.02 ± 0.26	0.15 ± 0.26	0.22 ± 0.25	−0.13 ± 0.26	0.36 ± 0.24	0.42 ± 0.22
L_S	−0.21 ± 0.25	−0.10 ± 0.26	−0.39 ± 0.23	0.08 ± 0.26	−0.37 ± 0.23	−0.22 ± 0.25
(Cont.)						
I^-	K_T	K_S	K_I	K_M	K_O	
D_T	−0.48 ± 0.18	0.17 ± 0.21	0.18 ± 0.21	0.16 ± 0.21	0.20 ± 0.20	
L_S	−0.15 ± 0.22	−0.17 ± 0.22	−0.16 ± 0.22	−0.16 ± 0.22	−0.16 ± 0.22	

5.4 Contributions of under-coordinated atoms are insignificant compared with facet orientations

In this work we attribute the main differences among regions, i.e., the corner/edge/flat facet regions of nanoplates, or the tips/side regions of nanorods, to the underlying facets – the proportion of Au{111} and Au{110}. We have considered another potential contribution to the difference, i.e., the under-coordinated atoms. The corner and edge regions of nanoplates are mainly exposing Au{110}; they also contain more under-coordinated atoms along the edges where the {111} and {110} facets meet compared with the flat facet regions, which expose Au{111}. On the contrary, the tip regions of nanorods (mainly Au{111}) have more under-coordinated atoms compared with the side regions (mainly Au{110}). For all ligands studied in this work, comparing the trends of different regions across nanoplates and nanorods, we found that *K* or *h* is dominated by the underlying facet instead of the under-coordinated atoms. For example, comparing Supplementary Table 3 (nanoplates) and Supplementary Table 6 (nanorods), $K_c^{\{110\}} > K_e^{\{110\}} > K_f^{\{111\}}$, $K_S^{\{110\}} > K_T^{\{111\}}$, $h_c^{\{110\}} < h_e^{\{110\}} < h_f^{\{111\}}$, and $h_S^{\{110\}} < h_T^{\{111\}}$ for CTAB. That is, no matter whether the {110} facets reside on the corners/edges of nanoplates (with more under-coordinated atoms) or on the sides of nanorods (with fewer under-coordinated atoms), they show a larger *K* and smaller *h* compared to {111} facets. Therefore, the underlying facets were considered as the main structural characteristics for ligand adsorption at different regions, between which the under-coordinated atoms have less significant contributions.

Of course, the differences of *K* and *h* in *sub-facet* regions, e.g., inner/middle/outer regions within the *same* flat facets of a nanoplate, are attributed to the differences in density of structural defects which are under-coordinated atoms compared with the regular facet atoms.

6 Supplementary control experiments and discussions on facet-controlled synthesis of Au nanoparticles demonstrating the crossover behavior of ligand adsorption

6.1 Ascorbic acid/ascorbate adsorption onto Au particles are likely insignificant in the presence of CTAB

Ascorbic acid is a commonly used mild reducing agent for the reduction of Au precursors during the synthesis of Au nanoparticles. Related to the discussion of the facet-controlled synthesis facilitated by the selective adsorption of ligands, we evaluated the potential adsorption of ascorbic acid and ascorbate on the Au surface, even though ascorbic acid and ascorbate were never discussed as a capping ligand in the literature, to the best of our knowledge.

In the reaction mixture of Au nanoparticle synthesis, the concentration of ascorbic acid is typically on the order of 0.1 – 1 mM, and pH is 3 – 5. The pK_a of ascorbic acid is 4.2, so both the protonated acid form (HA) and the deprotonated form (A^-) have a significant portion in the solution. We attempted to measure the adsorption affinity of HA and A^- on 5-nm Au nanoparticles via bulk COMPEITS titration. We found that upon mixing resazurin (R) and excess HA in water, R is readily reduced by HA without additional reducing agents or catalysts (Supplementary Fig. 21a). On the other hand, at pH 7.4 when [HA] is negligible compared to [A^-], very little change of absorbance of the mixture of R and A^- is observed over 18 min (Supplementary Fig. 21b), indicating that under basic conditions the direct reduction of R by A^- is

negligible even if thermodynamically favorable. In the reaction mixture consisting of R, NH_2OH, and 5-nm Au nanoparticles, higher [A^-] led to higher reaction rates (Supplementary Fig. 21c). In this case, ascorbate appears to act as a catalytic promoter phenomenologically. This catalytic rate promotion effect allows for the estimation of A^- adsorption affinity to Au nanoparticles through a concentration titration and fitting through an empirical kinetic saturation equation[24]:

$$v_0 = \frac{aK_{1/2}[\mathrm{L}]}{1 + K_{1/2}[\mathrm{L}]} + b \qquad \text{Eq. S21}$$

where $K_{1/2}$ is an apparent adsorption equilibrium constant and at [L] = $1/K_{1/2}$, the reaction rate reaches 50% of the maximum (saturation) rate. Fitting the titration curve of ascorbate (Supplementary Fig. 21c) gives $K_{1/2} \sim 6.4 \times 10^2$ M^{-1} for A^-, about three orders of magnitude smaller than K_{CTAB} (~6.0×10^5 M^{-1}). In addition, [CTAB] is higher than [HA] + [A^-] in a typical synthesis. Therefore, unless K_{HA} is much larger than K_{A^-} (which we believe is unlikely), the adsorption of ascorbate or ascorbic acid onto Au particles is probably insignificant compared with the adsorption of CTAB.

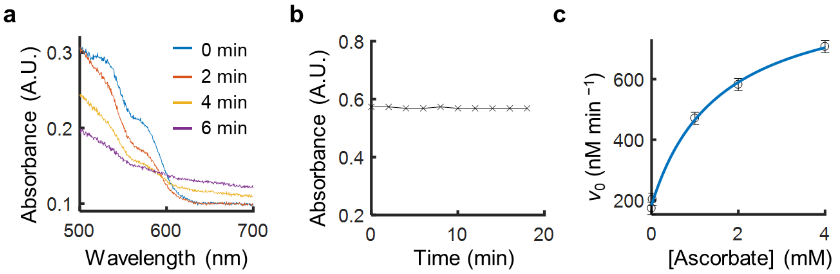

Supplementary Fig. 21 | Estimation of the binding affinity of ascorbic acid/ascorbate on 5-nm Au nanoparticles. a, Evolution of the reduction of R by AA in water, characterized by UV-Vis spectroscopy. $[R]_0$ = 10 μM, $[HA]_0 + [A^-]_0$ = 1.0 mM. **b**, Time evolution of the absorbance of the mixture of 10 μM of R and 1.0 mM of A^- in 7 mM pH 7.4 phosphate buffer at 604 nm (the maximum adsorption wavelength of R under basic conditions). **c**, Initial rate of the reduction of R as a function of [ascorbate]. Reaction conditions: $[R]_0$ = 10 μM, $[NH_2OH]_0$ = 1.0 mM, [Au nanoparticle] = 0.01 nM in 7 mM pH 7.4 phosphate buffer. Fitting of the curve gives $K_{1/2}$ = 0.64 ± 0.21 mM^{-1}, a = 0.72 + 0.18 μM/min, b = 0.18 + 0.02 μM/min.

6.2 Potential contribution of Au species adsorption in the facet-controlled Au nanoparticle synthesis

In the $HAuCl_4$ reduction by AA in the presence of CTAB to make Au nanoparticles, after the formation of nuclei, the growth of Au nanoparticles could come from the deposition of Au(0) atoms on the nuclei or existing nanoparticles. The Au(0) atoms could come from the growth solution, or could be generated from the reduction of adsorbed Au species, e.g., Au(I) or Au(III) species, on the nanoparticles[38,39]. We could not probe the adsorption of Au(I) or Au(III) species on Au nanoparticles using COMPEITS – these species are only stable in acidic conditions (the condition for Au nanoparticle synthesis) whereas the fluorogenic probe reaction for COMPEITS imaging used in this work requires slightly basic conditions, because the product molecule resorufin is only highly fluorescent in its deprotonated form.

Nevertheless, we rationalize that the potential preferential adsorption of Au species on different facets should have a minor role in shaping nanoparticles. If the Au species were to have significant preferences over a certain facet, one would not need to add additional stabilization ligand for shape control. The fact is that particles without a dominating facet is obtained if no stabilizer is used in the synthesis[5,40].

Above all, in our colloidal Au nanoparticle synthesis, the reactants ($HAuCl_4$ and ascorbic acid) are kept constant and only CTAB's concentration was varied to achieve different dominance of {111} vs. {110} facets on the resulting particles. It is reasonable to conclude that CTAB is the dominant player in controlling the surface facets, instead of other species in the solution; this conclusion is also consistent with many other studies of CTAB-controlled Au nanoparticle synthesis[5,38,39] .

6.3 The CTAB concentrations in Au nanoparticle syntheses are all below the critical micelle concentration at the reaction temperature

CTAB molecules are well-documented to aggregate and form micelles at concentrations above its critical micelle concentration (CMC). When the micelles appear, the number of free CTAB molecules in the solution will not be the same as that dissolved in the solution. In order to avoid the effect of micelle formation on CTAB adsorption, the Au nanoparticle syntheses were conducted at CTAB concentrations below the CMC. According to literature, the CMC of CTAB is strongly dependent on temperature, which can be depicted by a power law[41-43]:

$$\mathrm{CMC} \propto T^{n} \qquad \text{Eq. S22}$$

where the exponent $n > 1$. Supplementary Fig. 22 shows the temperature-dependent CMC of CTAB. At 30 ºC, the CMC of CTAB is 1.68 mM and increases to 2.15 mM at an elevated temperature of 45 ºC [43]. By extracting the points and fitting them based on a linear relationship, the CMC at 85 ºC (our synthesis reaction temperature) is derived to be 3.46 mM (Supplementary Fig. 22). It should be pointed out that compared with the power law ($n > 1$) shown in Eq. S21, the CMC of CTAB at 85 ºC derived from a linear relationship should be a lower estimate, meaning that the true CMC at 85 ºC should be greater than 3.46 mM. Regarding the Au nanoparticle syntheses that were conducted at 85 ºC, the highest [CTAB] was 3.12 mM, which is below 3.46 mM, the lower estimate of the CMC. Additionally, the crossover concentration of CTAB derived from our experiments is approximately 2 mM (Fig. 4j), much lower than the expected CMC at 85 ºC. Based on these results, we are confident to conclude that the effect of micelle formation of CTAB should be insignificant in our experiments.

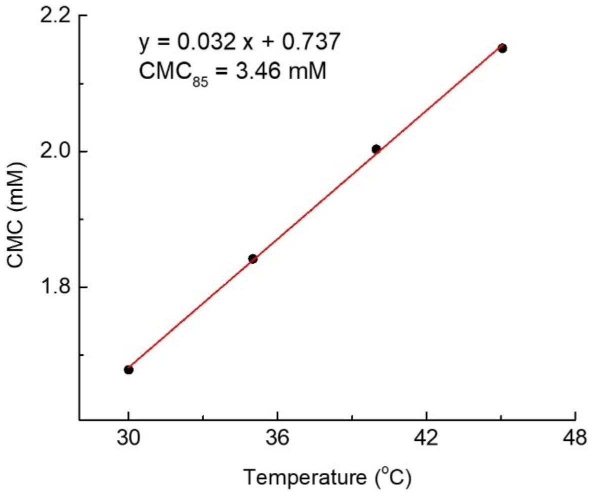

Supplementary Fig. 22 | Extrapolating the temperature-dependent critical micelle concentration of CTAB from the reported dependence on temperature. Based on a linear fitting of the points, the CMC of CTAB at 85 ºC is estimated as 3.46 mM. The data points were extracted from a reported study[43].

6.4 Possible reasons for the existence of crossover behavior of CTAB adsorption on Au{110} vs. Au{111}

Given $K^{\{110\}} > K^{\{111\}}$ for CTAB adsorption on Au surface as we determined in this study, the existence of crossover behavior of CTAB indicates $\rho_{\max}^{\{110\}} < \rho_{\max}^{\{111\}}$, meaning that the saturated adsorption density of CTAB on Au{110} is lower than that on Au{111}. One possible reason lies in the different surface packing density of Au atoms on different facets. The surface packing density of Au atoms on the {110} facets is noticeably lower than that on {111} facets (56% vs. 91%), which may result in a smaller number of sites for CTAB adsorption. Another possible contribution could originate from the facet-dependent adsorption configuration of CTAB. According to literature, both small and big molecules can have distinctive adsorption configurations on various facets[44,45]. One compelling example can be found in

the adsorption of F-containing peptide S7 (sequence, SSFPQPN; S, Serine; F, Phenylalanine; P, Proline; Q, Glutamine; N, Asparagine) on Pt surfaces, in which the peptide shows "lie-flat" and "stand-up" configurations on Pt{100} and Pt{111}, respectively[44]. The difference in adsorption configuration will then impose differentiable steric hindrance and thus affect the molecule adsorption. Nevertheless, our measurements cannot provide information on the molecular level adsorption configuration of CTAB on Au surfaces.

6.5 Predicting the crossover concentration c_x

One might predict whether a crossover concentration exists for two facets, and if yes, calculate the value of c_x. At the crossover concentration, $\rho^{strong} = \rho^{weak}$. On the basis of a non-cooperative Langmuir adsorption (assuming $h = 1$ for simplicity for Eq. 1), one can get

$$c_x = \frac{\rho_{max}^{strong} K^{strong} - \rho_{max}^{weak} K^{weak}}{K^{strong} K^{weak} (\rho_{max}^{weak} - \rho_{max}^{strong})} \qquad \text{Eq.S23}$$

It is worth noting that the adsorption equilibrium constant is typically dependent on temperature. Therefore, the predicted c_x will be temperature-dependent. Note for c_x to have a positive value, which is a prerequisite for the application of the cross-over concept for shape-controlled synthesis for other metals/materials, ρ_{max}^{weak} has to be greater than ρ_{max}^{strong}.

6.6 Potential broader applications of the crossover behavior of ligand adsorption

The crossover behavior of CTAB adsorption on Au{111} vs. Au{110} enabled us to control the facet distribution during Au nanoparticle synthesis by simply tuning [CTAB] in the solution. We envision that this crossover adsorption behavior of ligands on solid particles can potentially have other broader applications:

1) In controlled synthesis of nanoparticles
    a. Nanoparticle surface carving via selective etching: Here the ligand molecules can serve as an etchant, and by tuning the etchant concentration, one can selectively etch one facet vs. another.
    b. Ligand-induced galvanic replacement for the generation of various hollow structures: Here the ligand work as an agent to assist galvanic replacement, and turning the ligand can perhaps tune the replacement toward one specific facet.
    c. Facet-selective deposition on a solid particle: For example, one can selectively deposit metal onto semiconductor particles, or a second metal onto existing metal particles, while tuning the concentration of a ligand to vary the accessibility of respective facets.
2) In surface modification of nanoparticles
    a. Surface functionalization: one can use a ligand to change the relative accessibility of two different facets and then add a functionalization reagent to modify preferentially one facet vs. the other.
    b. Ligand exchange: One can selectively exchange ligands on one facet to change the surface property, for example, from hydrophilic to hydrophobic.
3) In heterogeneous catalysis
    a. Product selectivity control: If different facets of a catalyst have different product selectivity, one might tune the ratio of different products via tuning the concentration or partial pressure of the reactant, or tuning the concentration or partial pressure of a ligand that blocks one facet.
    b. Catalyst poisoning mitigation and thus durability improvement: One can tune the concentration of a reagent to slow down the generation rate of a poisonous intermediate or product on one facet, leading to prolonged usage of the catalyst.

6.7 Predicting relative multi-layer adsorption trends

Our estimation of the density of adsorbed ligands from Eq. 2 is applicable in the regime of monolayer adsorption, as it is based on the Langmuir adsorption model on which the Hill model of cooperativity is added. Consistently, our COMPEITS imaging specifically probes the first-layer adsorption, because multi-layer adsorption does not provide further suppression of the fluorogenic auxiliary reaction rate. However, the ligands we studied here, including CTAB, could potentially have multi-layer adsorption on Au surfaces. Nevertheless, the monolayer adsorption scenario as in Eq 2 likely still offers useful predictions on the relative adsorption density of the same ligand on two different surfaces under the same conditions, e.g., the adsorption of CTAB on different Au facets. The key differences of the multi-layer adsorption from the monolayer adsorption are the stacking of ligands in the dimension perpendicular to the surface and the resultant intermolecular interactions of these ligands. Therefore, if one ligand shows a larger adsorption density on one surface over another under monolayer adsorption conditions, the same preference should preserve in multi-layer adsorption, because the intermolecular interactions of ligands in the perpendicular dimension should be comparable on different surfaces, unless long-range interactions between the ligand and the surfaces occur.

6.8 The crossover behavior in our shape-controlled synthesis of Au nanoparticles should not be caused by the seeding effect

The geometry differences from synthesis with varying [CTAB] are attributed to the ligand adsorption behaviors throughout the synthesis time rather than from the initial seeding. Our synthesis was a one-pot approach and does not involve the use of pre-formed seeds, but we understand that nuclei, also called seeds, could still be in situ generated during the nucleation process of a one-pot synthesis. The type of seeds could potentially affect the shape taken by a product particle because the internal structure (e.g., single-crystal vs. twinned structure) could somewhat constrain the shape expression of nanocrystals. However, for nanocrystals growing from the same seeds (i.e., same internal structure), they can still be diverse in shapes depending on the properties of a capping agent or facet directing reagent. For example, single-crystal seeds can grow into cubes and octahedra; penta-twinned seeds can grow into decahedra and nanorods; planer-defect seeds can grow into nanoplates and nanocubes[46]. All these examples of distinctive pairs of particle products are characterized by both different shapes and different facets, despite the same internal structure. Therefore, the crossover behavior in the facet distribution of our synthesis with varying [CTAB] should stem from the ligand adsorption rather than the seeding effect.

7 Supplementary references

1. Shankar, S. S. *et al.* Biological synthesis of triangular gold nanoprisms. *Nat. Mater.* **3**, 482-488 (2004).
2. Andoy, N. M. *et al.* Single-molecule catalysis mapping quantifies site-specific activity and uncovers radial activity gradient on single 2D nanocrystals. *J. Am. Chem. Soc.* **135**, 1845-1852 (2013).
3. Kan, C., Wang, G., Zhu, X., Li, C. & Cao, B. Structure and thermal stability of gold nanoplates. *Appl. Phys. Lett.* **88**, 071904 (2006).
4. Xie, J., Lee, J. Y. & Wang, D. I. C. Synthesis of single-crystalline gold nanoplates in aqueous solutions through biomineralization by serum albumin protein. *J. Phys. Chem. C* **111**, 10226-10232 (2007).
5. Grzelczak, M., Pérez-Juste, J., Mulvaney, P. & Liz-Marzán, L. M. Shape control in gold nanoparticle synthesis. *Chem. Soc. Rev.* **37**, 1783-1791 (2008).
6. Wang, C. *et al.* Synthesis of high-yield gold nanoplates: fast growth assistant with binary surfactants. *J. Nanomater.* **2010**, 969030 (2010).
7. Zhou, X. *et al.* Quantitative super-resolution imaging uncovers reactivity patterns on single nanocatalysts. *Nat. Nanotechnol.* **7**, 237-241 (2012).
8. Botella, P., Corma, A. & Navarro, M. T. Single gold nanoparticles encapsulated in monodispersed regular spheres of mesostructured silica produced by pseudomorphic transformation. *Chem. Mater.* **19**, 1979-1983 (2007).

9. Stöber, W., Fink, A. & Bohn, E. Controlled growth of monodisperse silica spheres in the micron size range. *J. Colloid Interface Sci.* **26**, 62-69 (1968).
10. Liz-Marzán, L. M., Giersig, M. & Mulvaney, P. Synthesis of Nanosized Gold−Silica Core−Shell Particles. *Langmuir* **12**, 4329-4335 (1996).
11. Aliaga, C. *et al.* Sum frequency generation and catalytic reaction studies of the removal of organic capping agents from Pt nanoparticles by UV−ozone treatment. *J. Phys. Chem. C* **113**, 6150-6155 (2009).
12. Scarabelli, L., Sánchez-Iglesias, A., Pérez-Juste, J. & Liz-Marzán, L. M. A "Tips and Tricks" Practical Guide to the Synthesis of Gold Nanorods. *J. Phys. Chem. Lett.* **6**, 4270-4279 (2015).
13. Wang, Z. L., Gao, R. P., Nikoobakht, B. & El-Sayed, M. A. Surface reconstruction of the unstable {110} surface in gold nanorods. *J. Phys. Chem. B* **104**, 5417-5420 (2000).
14. Gai, P. L. & Harmer, M. A. Surface atomic defect structures and growth of gold nanorods. *Nano Lett.* **2**, 771-774 (2002).
15. Johnson, C. J., Dujardin, E., Davis, S. A., Murphy, C. J. & Mann, S. Growth and form of gold nanorods prepared by seed-mediated, surfactant-directed synthesis. *J. Mater. Chem.* **12**, 1765-1770 (2002).
16. Jana, N. R., Gearheart, L. & Murphy, C. J. Wet chemical synthesis of high aspect ratio cylindrical gold nanorods. *J. Phys. Chem. B* **105**, 4065-4067 (2001).
17. Wuttig, A., Yaguchi, M., Motobayashi, K., Osawa, M. & Surendranath, Y. Inhibited proton transfer enhances Au-catalyzed CO_2-to-fuels selectivity. *Proc. Natl. Acad. Sci. U.S.A.* **113**, E4585-E4593 (2016).
18. Herrero, E., Buller, L. J. & Abruña, H. D. Underpotential deposition at single crystal surfaces of Au, Pt, Ag and other materials. *Chem. Rev.* **101**, 1897-1930 (2001).
19. Hill, A. V. The possible effects of the aggregation of the molecules of haemoglobin on its dissociation curves. *J. Physiol. (Lond.)* **40**, 4-7 (1910).
20. Villarreal, E., Li, G. G., Zhang, Q., Fu, X. & Wang, H. Nanoscale Surface Curvature Effects on Ligand–Nanoparticle Interactions: A Plasmon-Enhanced Spectroscopic Study of Thiolated Ligand Adsorption, Desorption, and Exchange on Gold Nanoparticles. *Nano Lett.* **17**, 4443-4452 (2017).
21. Cornish-Bowden, A. *Fundamentals of enzyme kinetics*. (John Wiley & Sons, 2013).
22. Mao, X., Liu, C., Hesari, M., Zou, N. & Chen, P. Super-resolution imaging of non-fluorescent reactions via competition. *Nat. Chem.* **11**, 687-694 (2019).
23. Xu, W., Kong, J. S., Yeh, Y.-T. E. & Chen, P. Single-molecule nanocatalysis reveals heterogeneous reaction pathways and catalytic dynamics. *Nat. Mater.* **7**, 992-996 (2008).
24. Zou, N. M. *et al.* Cooperative communication within and between single nanocatalysts. *Nat. Chem.* **10**, 607-614 (2018).
25. Chen, T.-Y. *et al.* Concentration- and chromosome-organization-dependent regulator unbinding from DNA for transcription regulation in living cells. *Nat. Commun.* **6**, 7445 (2015).
26. Sambur, J. B. *et al.* Sub-particle reaction and photocurrent mapping to optimize catalyst-modified photoanodes. *Nature* **530**, 77-80 (2016).
27. Thompson, R. E., Larson, D. R. & Webb, W. W. Precise Nanometer Localization Analysis for Individual Fluorescent Probes. *Biophys. J.* **82**, 2775-2783 (2002).
28. Rust, M. J., Bates, M. & Zhuang, X. Sub-diffraction-limit imaging by stochastic optical reconstruction microscopy (STORM). *Nat. Methods* **3**, 793-796 (2006).
29. Bates, M., Huang, B., Dempsey, G. T. & Zhuang, X. Multicolor Super-Resolution Imaging with Photo-Switchable Fluorescent Probes. *Science* **317**, 1749-1753 (2007).
30. Chen, S. *et al.* Rapid seedless synthesis of gold nanoplates with microscaled edge length in a high yield and their application in SERS. *Nanomicro. Lett.* **8**, 328-335 (2016).
31. Meena, S. K. *et al.* The role of halide ions in the anisotropic growth of gold nanoparticles: a microscopic, atomistic perspective. *Phys. Chem. Chem. Phys.* **18**, 13246-13254 (2016).
32. Rubinstein, M. & Colby, R. H. in *Polymer Physics* 114 (Oxford University Press).

33. Ghosh, S. & Manna, L. The many "facets" of halide ions in the chemistry of colloidal inorganic nanocrystals. *Chem. Rev.* **118**, 7804-7864 (2018).
34. Magnussen, O. M. Ordered anion adlayers on metal electrode surfaces. *Chem. Rev.* **102**, 679-726 (2002).
35. Almora-Barrios, N., Novell-Leruth, G., Whiting, P., Liz-Marzán, L. M. & López, N. Theoretical description of the role of halides, silver, and surfactants on the structure of gold nanorods. *Nano Lett.* **14**, 871-875 (2014).
36. Ravi, V., Binz, J. M. & Rioux, R. M. Thermodynamic profiles at the solvated inorganic–organic interface: The case of gold–thiolate monolayers. *Nano Lett.* **13**, 4442-4448 (2013).
37. Schessler, H. M., Karpovich, D. S. & Blanchard, G. J. Quantitating the balance between enthalpic and entropic forces in alkanethiol/gold monolayer self assembly. *J. Am. Chem. Soc.* **118**, 9645-9651 (1996).
38. Yang, T.-H. *et al.* Toward a quantitative understanding of the reduction pathways of a salt precursor in the synthesis of metal nanocrystals. *Nano Lett.* **17**, 334-340 (2017).
39. Yang, T.-H., Gilroy, K. D. & Xia, Y. Reduction rate as a quantitative knob for achieving deterministic synthesis of colloidal metal nanocrystals. *Chem. Sci.* **8**, 6730-6749 (2017).
40. Lohse, S. E. & Murphy, C. J. The quest for shape control: A history of gold nanorod synthesis. *Chem. Mater.* **25**, 1250-1261 (2013).
41. Zieliński, R., Ikeda, S., Nomura, H. & Kato, S. Effect of temperature on micelle formation in aqueous solutions of alkyltrimethylammonium bromides. *J. Colloid Interface Sci.* **129**, 175-184 (1989).
42. Gonzalez-Perez, A. & Sanchez-Dominguez, M. Changes in self-assemblies induced by temperature, concentration and light. *Front. Biosci.* **5**, 611-630 (2013).
43. Cookey, G. A. & Obunwo, C. C. Effects of sodium bromide salt and temperature on the behaviour of aqueous solution of cetyltrimethylammonium bromide. *IOSR J. Appl. Chem.* **7**, 34-38 (2014).
44. Ruan, L. *et al.* Tailoring molecular specificity toward a crystal facet: a lesson from biorecognition toward Pt{111}. *Nano Lett.* **13**, 840-846 (2013).
45. Liu, P., Qin, R., Fu, G. & Zheng, N. Surface coordination chemistry of metal nanomaterials. *J. Am. Chem. Soc.* **139**, 2122-2131 (2017).
46. Shi, Y. *et al.* Noble-Metal Nanocrystals with Controlled Shapes for Catalytic and Electrocatalytic Applications. *Chem. Rev.* **121**, 649-735 (2021).
