# Supplementary material for: Nanoscale cooperative adsorption for materials control
Source: Nat Commun. 2021 Jul 13;12:4287. doi: 10.1038/s41467-021-24590-y (PMC8277846; doi:10.1038/s41467-021-24590-y)
Supplement: Supplementary file 3 — Description of Additional Supplementary Files [file 41467_2021_24590_MOESM3_ESM.pdf]

Description of Additional Supplementary Files

File Name: Supplementary Software 1
Description: MatLab codes for data analysis
