# Supplementary material for: Nanoscale cooperative adsorption for materials control
Source: Nat Commun. 2021 Jul 13;12:4287. doi: 10.1038/s41467-021-24590-y (PMC8277846; doi:10.1038/s41467-021-24590-y)
Supplement: Supplementary file 4 — Supplementary Software 1 [file 41467_2021_24590_MOESM4_ESM.zip › codes_sharing/ReadMe_NComm_16Dec20.pdf]

Introduction for **codes_sharing** folder

This **codes_sharing** folder includes MATLAB codes related to the manuscript "Nanoscale cooperative adsorption for materials control" by Rong Ye[†], Ming Zhao[†], Xianwen Mao, Zhaohong Wang, Diego A. Garzón, Heting Pu, Zhiheng Zhao, Peng Chen*.

The .m files in this folder are scripts for data analysis by MATLAB. The data output from each script is either saved by the script or manually exported from the Workspace of MATLAB. The analysis procedures are described in Supplementary Information section 1.8 of the manuscript.

The **s_iqPALM** folder contains a package of codes collectively named the 'subtraction iqPALM', which was expanded from iqPALM (*1, 2*) and whose major steps of data analysis were described and employed in ref (*3*). The original fluorescence movies were first processed with subtraction iqPALM for single-molecule fluorescence image analysis for super-resolution localization, as detailed in Supplementary Information section 1.8.1. In our routine data analysis, we first used 'Mao_create_mask_FLimage.m' to choose areas of interest and position markers, then used 'Mao_SMforGOLD_main.m' to obtain fitting results.

dissect_get_rate.m contains several functionalities: 1) filter the PSF-fitted single-molecule localizations based on pre-set parameters to correct for over-counting and underestimation of product molecules (see details in Supplementary Information section 1.8.2); 2) sort the filtered single-molecule localizations into different regions on the catalyst particle; 3) calculate the reaction rate for each region; and 4) obtain the adsorption parameters such as K and h via data fitting.

Fil_analy.m loads the output from the (subtraction) iqPALM, filters the fitted events based on various parameters (e.g., intensity, sigma x, etc) in the range set by the user, and generates 9 plots for facile visualization of the data. These 9 plots are: 1) a heat map of event coordinates (x and y in nm), 2) the scatter plot of event coordinates (x and y in nm), 3) the histogram of events based on the frame index, 4) the heat map of sigma x and sigma y, 5) the histogram of sigma x, 6) the histogram of Error x, 7) the heat map of sigma x and intensity, 8) the scatter plot of sigma x vs. intensity, and 9) the histogram of intensity. This script contains the calculation of localization error (i.e., Error x and Error y). **np_extract.m** allows the user to confine the events to look at within a given range in space (x and y in nm).

OverlayOMSEM_Part1.m and **OverlayOMSEM_Part2.m** transform coordinates from an optical microscopy (OM) image to the coordinates in a scanning electron microscopy (SEM) image.

plate_edge_detection.m detects the edge of nanoplates from an SEM image and extract their coordinates.

plate_fit_edge.m fits the edges of nanoplates with a linear function and calculate the coordinates of the vertices.

rod_edge_detection.m detects the edge of rods from an SEM image and extract their coordinates.

rods_fit_edge.m models the tips and side of a nanorod and gets coordinates of some key points.

References

1. P. Chen, T.-Y. Chen, MATLAB code package: iQPALM (image-based quantitative photo-activated localization microscopy), doi.org/10.6084/m9.figshare.12642617.v1 (2020).
2. T.-Y. Chen *et al.*, Concentration- and chromosome-organization-dependent regulator unbinding from DNA for transcription regulation in living cells. *Nat. Commun.* **6**, 7445 (2015).
3. X. Mao, C. Liu, M. Hesari, N. Zou, P. Chen, Super-resolution imaging of non-fluorescent reactions via competition. *Nat. Chem.* **11**, 687-694 (2019).
